# A Secure and Energy-Efficient Cross-Layer Network Architecture for the Internet of Things

**DOI:** 10.3390/s25113457

**Published:** 2025-05-30

**Authors:** Rashid Mustafa, Nurul I. Sarkar, Mahsa Mohaghegh, Shahbaz Pervez, Robert Morados

**Affiliations:** 1 Department of Computer and Information Sciences, Auckland University of Technology, Auckland 1010, New Zealand; rashid.mustafa@autuni.ac.nz (R.M.); mahsa.mohaghegh@aut.ac.nz (M.M.); 2 School of Information Technology, Whitecliffe College of Arts and Design, Auckland 1010, New Zealand; shahbazp@whitecliffe.ac.nz (S.P.); 20231097@mywhitecliffe.com (R.M.)

**Keywords:** cross-layer network architecture, internet of things, secure IoT, energy efficient

## Abstract

A secure and energy-efficient network architecture is essential due to the rapid proliferation of Internet of Things (IoT) devices in critical sectors such as healthcare, smart cities, and industrial automation. In this paper, we propose a secure and energy-efficient cross-layer IoT architecture. The security features and energy-saving techniques across various open-system interconnected protocol layers are incorporated in the proposed architecture. The improved security and energy efficiency are achieved using the lightweight Speck and Present ciphers, as well as adaptive communication strategies. The system performance is evaluated by testbeds and extensive simulation experiments using Cooja (Contiki operating systems) and NS-3. The simulation results obtained show a 95% attack mitigation effectiveness, 30% reduction in energy usage, and 95% packet delivery ratio. In a 20-node network scenario, Speck uses 5.2% less radio power than the Advanced Encryption Standard (AES), making it the best tradeoff among the investigated encryption techniques. The NS-3 simulation results confirm that lightweight encryption, such as Present and Speck, uses much less power than the traditional AES, which makes them more appropriate for IoT contexts with limited energy. The scalability across various IoT contexts is ensured through a hybrid assessment approach that combines hardware testbeds and simulation for system validation. Our research findings highlight opportunities for advancing IoT systems toward secure and energy-efficient smart ecosystems.

## 1. Introduction

By facilitating smooth connectivity between billions of devices in vital areas such as healthcare, smart cities, and industrial automation, the IoT has completely transformed contemporary computing. However, balancing strong security and an energy economy is extremely difficult due to the resource-constrained nature of IoT devices, including limited computing power, battery life, and memory. Although effective, traditional security measures, such as the Advanced Encryption Standard (AES-128), have prohibitive computational and energy overheads, which makes them unfeasible for IoT nodes that run on batteries. However, lightweight cryptographic algorithms (such as the Present cipher) save energy but frequently compromise cryptographic resilience, leaving networks vulnerable to brute-force or side-channel attacks. The current cross-layer architectures make an effort to resolve these trade-offs, but they are still disjointed and concentrate on security or energy optimization instead of balancing the two. The dynamic network conditions are often overlooked by standard cross-layer frameworks, whereas protocols such as RPL/6LoWPAN optimize the routing efficiency without adaptive encryption. Additionally, previous research has mainly relied on theoretical models or simulations, ignoring validation across heterogeneous contexts (such as scalable networks and hardware testbeds), resulting in solutions that do not work in real-world deployments.

This study proposes a cross-layer IoT architecture that addresses these issues by incorporating adaptive security and energy optimization across an open systems interconnection (OSI) model. In contrast to other frameworks, our proposal achieves a 30% reduction in power consumption without sacrificing security by introducing a dynamic encryption engine that dynamically shifts between AES, Speck, and Present according to the real-time network traffic and energy availability. We integrated Cooja/Contiki simulations, hardware platforms (Z1, EXP430F5438), and mathematical models to solve the lack of hybrid validation in IoT research, guaranteeing scalability (up to 20 nodes) and practical applicability. The proposed method advances beyond the existing ones, which are briefly discussed below. First, it offers the highest level of security and consumes less energy because of the use of the Present and Speck lightweight ciphers. While the Present cipher proves to be the most efficient solution in terms of overall power consumption, Speck provides a balanced approach, making it well suited for resource-constrained environments. Second, the proposed system integrates the adaptive security algorithms at various OSI layers (cross-layer), enabling dynamic trade-offs between encryption strength and energy consumption, unlike the traditional ELITE [1], which works on MAC-layer energy optimization. The proposed method circumvents the computational cost of blockchain by utilizing cross-layer, lightweight, and context-aware encryption. Unlike the traditional AI/blockchain and crypto efforts that rely on fixed algorithms, our dynamic engine chooses the best ciphers (AES/Speck/Present) in real time using a mix of simulation and hardware testing. According to our research, Speck is one of the best lightweight ciphers for the IoT because it uses 37% less CPU power and 5.2% less radio power than the AES in large-scale networks. Furthermore, we focus on the practical aspects of IoT applications that are lacking in previous studies. However, we assess the protocol behavior and energy consumption at the cross-layer, such as the application, network, and sensor layers, using the NS-3.42 simulation environment. With the support of IPv6, UDP communication, flow monitoring, and custom power logging, NS-3 makes it possible to test IoT configurations in a modular and scalable manner. This makes it possible to precisely examine the effects of encryption schemes such as AES, Speck, and Present on system performance in simulated real-time scenarios. For instance, we avoid using MQTT in sensitive applications and prioritize hierarchical topologies. This design strategy addresses a long-standing gap in cross-layer real-world IoT frameworks by resolving the crucial trade-off between security and sustainability, advancing IoT systems toward more robust and energy-efficient smart ecosystems.

### 1.1. Research Challenges

The IoT devices are rapidly proliferating in vital areas, such as healthcare and smart cities, necessitating architectures that balance energy efficiency and security. However, there are many research obstacles due to the diverse characteristics of IoT ecosystems, including resource-constrained devices, dynamic network conditions, and changing cyberthreats. These research challenges and questions are highlighted next.

- Research Question 1: What cross-layer IoT architecture can be developed for a secure and energy-efficient network?
  To address this question, a thorough review and analysis of cross-layer IoT frameworks for secure and energy-efficient networks was carried out. To test and mitigate man-in-the-middle (MitM), eavesdropping, data manipulation, and application layer vulnerabilities, the real-world data collected by Contiki was compared with the Cooja 2.7 Virtual IoT Simulator. Furthermore, robust security was tested to see whether regular firmware updates and network segmentation were necessary in addition to implementing Transport Layer Security (TLS) using the COAP protocol. By simulating sensor behavior in controlled settings, this study sheds light on how well these networks perform in practical settings.
- Research Question 2: What cross-layer secure protocol can be developed for IoT applications?
  This question is answered by proposing a cross-layer framework that improves security and energy efficiency for Internet of Things applications. The main goals of the framework are to ensure the availability, confidentiality, integrity, and privacy of data across various layers of the IoT network. The promotion of energy-efficient algorithms, the identification of unique IoT security threats, the guarantee of system scalability, and the promotion of compatibility and interoperability are all important factors. In addition, the framework supports environmental sustainability objectives, which broaden the scope of IoT applications and make the ecosystem secure and effective.
- Research Question 3: What metrics can be used to quantify the security and energy efficiency of the proposed architecture?
  The effectiveness of the proposed architecture was evaluated using various performance measures, including energy consumption, packet delivery ratio, network throughput, latency, and security vulnerabilities, to address Research Question 3. These metrics are crucial in assessing the energy efficiency and security of an IoT system. Specifically, energy consumption was measured by analyzing the power usage of devices during transmission and processing. The packet delivery ratio and network throughput are important indicators of data transmission performance in an IoT system. In real-time applications, latency is a critical metric that measures the delay in data transmission. The security of the system was evaluated using simulations and a testbed, assessing its ability to defend against threats such as data manipulation, eavesdropping, and man-in-the-middle (MitM) attacks. To evaluate the performance of protocols like LEACH, RPL, and ContikiMAC, we utilized both real-world and simulated data from the Contiki Cooja 2.7 Virtual IoT simulator. By realistically simulating sensor behavior, this study provides a comprehensive evaluation of the architecture’s ability to balance security and energy usage in real-world IoT deployments.

### 1.2. Study Contribution

The primary contribution of this study is the design, simulation, and validation of a cross-layer architecture for energy-efficient and secure IoT networks, which enhances the realization of sustainable and secure smart environments. This paper provides important insights into energy-efficient protocols, lightweight cryptography, and cross-layer security architectures in resource-constrained IoT ecosystems. The effectiveness and performance of the suggested framework can be evaluated under a variety of cyber threat scenarios and with varying node counts, thanks to the use of the Contiki/Cooja simulator and real-world data analysis. In particular, the proposed cross-layer architecture balances strong security measures with the limited resources available in various IoT deployments. This advancement, along with system analysis, significantly improves the practical applications of secure and energy-efficient IoT systems.

The main contributions of this study are summarized as follows:

- We develop a cross-layer IoT architecture that addresses the crucial equilibrium between energy efficiency and security, providing a thorough understanding of key factors that can be co-optimized in various IoT application scenarios. To this end, we configure a Contiki/Cooja simulator for system performance analysis and evaluation of the viability and efficiency of various energy-saving and security measures in intricate IoT environments.
- We propose energy-efficient routing protocols and lightweight cryptography to support the proposed cross-layer IoT architecture. To this end, we examined the performance of energy-efficient routing protocols and lightweight cryptographic protocols (AES, Speck, and Present ciphers) in the context of the IoT. We improve the security and sustainability of IoT networks, showcasing their useful applications in striking a balance between resource consumption and security requirements.
- We analyze a comprehensive simulation-based assessment using Cooja and NS-3, connecting the theoretical analysis with real-world validation, including hardware testbed measurements on the Z1 and EXP430F5438 platforms. Finally, we validate Cooja Contiki results by NS-3 simulation, as well as LrWpanHelper and SixLowPanHelper.

## 2. Related Work

Although several studies have suggested energy-efficient objective functions (OFs) for IoT routing, most of them have focused only on the routing layer, generally ignoring the crucial role that the dynamics of the Medium Access Control (MAC) layer play in overall energy performance. To overcome this restriction, Reference [1] presented ELITE, a cross-layer OF that incorporates the strobe per packet ratio (SPR) to take MAC-layer radio duty cycling (RDC) regulations into account. ELITE prioritizes routes with fewer strobe transmissions, resulting in up to 39% less energy usage in IoT nodes. This shows how cross-layer optimization can improve network lifespan. The problem of balancing security and power efficiency in IoT systems is examined in this study. Our proposed cross-layer architecture combines network, application, and physical layer protocols to improve energy efficiency and defend against cyberattacks [2]. Validating the performance with Cooja simulations provides insightful information for designing IoT systems with limited resources. A thorough examination of secure and energy-efficient cross-layer IoT frameworks is provided in this survey, which highlights the shortcomings of conventional security techniques and promotes blockchain integration and AI-powered intrusion detection [3]. This emphasizes the importance of IoT systems to have multiple layers of security and sustainability, especially when developing smart city infrastructures in industrial and agricultural settings. The integration of the IoT into critical infrastructures, such as the healthcare, finance, and energy sectors, has raised concerns regarding the security of user privacy and data integrity. An isolation forest algorithm was used to protect the classifiers from data poisoning, and ML classifiers were used to differentiate between attack and non-attack data in an architecture based on onion routing and machine learning to reduce these risks [4]. The onion routing network ensures triple encryption of IoT data, with blockchain nodes verifying the data, resulting in enhanced security and lower computational costs compared with traditional methods, achieving 97.7% accuracy with a Support Vector Machine (SVM). The necessity for improved security measures to shield embedded devices from potential attacks has been highlighted by the expanding use of these devices in the IoT ecosystem [5]. To guarantee secure firmware version verification and integrity validation, this study proposes a firmware update architecture that combines blockchain technology with a Physical Unclonable Function (PUF). The proposed framework solves vulnerabilities in out-of-date firmware and offers a defence against attacks that target known firmware flaws by utilizing the PUF’s challenge–response pairs for device authentication and blockchain for secure firmware upgrades. The rapid expansion of the IoT across various sectors has led to significant data privacy and security challenges, as traditional access control solutions are often vulnerable to single points of failure. This study introduced a decentralized, blockchain-integrated framework that incorporates an accredited access control scheme, attribute-based cryptography, and smart contracts to enhance security and privacy [6]. The proposed framework mitigates DoS attacks, improves data protection, and outperforms previous approaches, achieving high accuracy (96.9%), precision (98.43%), and recall (98.43%), thereby demonstrating its effectiveness in securing IoT systems. In resource-constrained contexts, such as the IoT and CPS, modern machine learning and artificial intelligence models, including Deep Neural Networks (DNNs) and Large Language Models (LLMs), encounter issues related to energy conservation, security, and reliability despite achieving great accuracy in a variety of applications [7]. To create reliable, energy-efficient ML systems for EdgeML and tinyML applications, this review investigates cross-layer optimization strategies in hardware and software. To improve the secure and scalable implementation of AI, new developments in multimodal LLMs, continuous learning, and quantum machine learning have also been covered. In this review, the grasshopper optimization algorithm (GOA), Bat Algorithm (BA), and whale optimization algorithm (WOA) were combined with K-means and a new cost function to investigate energy-efficient clustering in WSNs [8]. According to the results, the WOA operates better in complicated contexts by maximizing energy use and prolonging network lifetime, whereas the the GOA performs best in simple scenarios. This study emphasizes how clustering and optimization can improve WSN efficiency, particularly in environments with restricted resources. This review explores the convergence of the IoT, AI, and blockchain for data integration at the edge, addressing challenges such as interoperability, security, and scalability. The proposed DENOS model extends traditional architectures with semantic and convergence layers, enabling secure cross-domain collaboration and data-driven decision making. The integration of blockchain, the IoT, and AI in Beyond 5G (B5G) networks is examined in this review, which addresses the issues of scalability, security, and connection in next-generation networks (NGNs) [9]. The SDN and federated blockchains are used in the proposed Secure Interconnected Autonomous System Architecture (SIASA) to provide secure data exchange, decentralization, and interoperability across multidomain IoT ecosystems. The SHA-256 algorithm and six general-purpose input/output (GPIO) pins were integrated into this review’s secure SoC architecture for the IoT to improve data security and confidentiality [10]. For IoT security applications, the proposed design strikes a good mix of hardware adaptability, battery efficiency, and cryptographic robustness. The scalability and privacy issues of a blockchain-based distributed system for secure and effective Industrial Internet of Things (IIoT) data exchange are examined in this study [11]. The proposed architecture strengthens access control, lowers latency, and improves data management in industrial contexts by combining edge computing with a smart-contract framework. With the integration of a Two-Fold Physically Unclonable Function (TF-PUF) and Montgomery Curve Encryption (MCE) for authentication and Hybrid Start Peer-to-Peer (HyS-P2P) topology for resource allocation, a novel secure triune layered (Sec-TriL) architecture for secure task management in the fog-assisted IoT is examined in this study [12]. The proposed method, which is assessed using the iFogSim simulator, optimizes energy, security, and efficiency by leveraging intelligent job offloading and adaptive scheduling. In contrast to blockchain, this analysis examines an IoT microservice architecture built on Holochain that improves security, scalability, and energy efficiency [13]. According to the results, Holochain is a good substitute for IoT networks because it lowers energy usage by more than 60% and boosts performance by 50%. This review examines ChainFL, a federated learning system powered by blockchain that improves the scalability and effectiveness of edge computing for the IoT [14]. ChainFL improves training efficiency by 14% compared to conventional FL systems by incorporating DAG-based mainchain and subchain sharding.

This review examines the SAR-CRN architecture, which uses cognitive radio to facilitate effective spectrum sharing and improve IoT security and dependability [15]. In secure communication for resource-constrained IoT contexts, the proposed relay-selection technique outperforms traditional CR networks in terms of secrecy and decoding performance. By proposing a vertical IoT framework (edge–fog–cloud) for e-commerce and integrating machine learning algorithms such as CNNs, NLU, and RL to optimize customized production, consumer behavior analysis, and decision-making in dynamic smart environments, this study bridges the gap between Industry 4.0 and Industry 5.0 [16]. Economic sustainability and adaptive e-commerce models are advanced by aligning ML-driven solutions with vertical IoT architectures, addressing gaps in scalable, personalized consumer–company interactions and Industry 5.0’s demand for decentralized, AI-enhanced economic ecosystems. This study tackles important but little-studied flaws in the TCP/IP protocol suite’s cross-layer interactions, namely faked ICMP error signals that allow off-path attackers to take advantage of systemic weaknesses even when individual protocols are resilient [17]. This study emphasizes the necessity of integrated, cross-layer security frameworks to reduce systemic hazards in contemporary network topologies by exposing the ways in which seemingly secure protocols combine to produce exploitable risks. To improve the latency efficiency and performance metrics, this study proposes a CDML framework that uses demand-density optimization and security assessments to overcome data reliability and security challenges in IoT edge computing. This framework outperforms current approaches in robust distributed data management [18]. The framework advances scalable solutions for reliable IoT edge ecosystems by combining dynamic request–response categorization with stringent security validation. This study provides high-confidence resource allocation and reduces the risks in decentralized networks. Another study proposed AIMS, an intrusion detection system that combines a Cooja-simulated AMI-RPL Attack Dataset (ARAD), a stacked ensemble model, and spider monkey optimization-based feature selection to predict and mitigate attacks to solve RPL security vulnerabilities in IoT-driven smart grids [19]. AIMS offers a strong framework for protecting AMI deployments in resource-constrained situations by combining lightweight blockchain detection and cryptocurrency-driven isolation, resulting in increased attack resistance (high prediction accuracy) and an extended network lifetime. The current and prospective IoT architectures are compared in Table 1. The development of the IoT from a technological and sociological standpoint is summarized in this thorough overview, which also analyses key issues with security, scalability, and interoperability across smart ecosystems, and suggests a tiered design with enabling technologies [20]. Market trends, functional blocks, and simulation tools are evaluated to identify unmet requirements and provide researchers with a path for addressing the socio-technical challenges of the IoT and advancing scalable and sustainable implementations. Real-time environmental monitoring is made possible by Wireless Sensor Networks (WSNs) through multihop communication; nevertheless, a major obstacle is still ensuring that data aggregation is secure and energy-efficient [21]. The proposed Secure Cluster-Based Data Aggregation Protocol (SCDAP) aims to address this issue by improving security through effective key generation and authentication while reducing transmission overhead and energy usage. IoT integration improves sustainability and efficiency in smart renewable energy systems, but it also exposes them to serious cybersecurity risks, such as denial-of-service attacks and fake data injection [22]. This study emphasizes the urgent need for strong grid security measures by highlighting the serious flaws in authentication and communication methods. Machine learning is essential for improving automation and security in restructured enterprise architectures that are required for the integration of the IoT with enterprise systems [23]. While stressing the necessity for targeted research on ML-driven IoT solutions, this study highlights ML’s ability to evaluate IoT data, identify abnormalities, and optimize smart operations. With the rise of multimedia and interactive services in 5G and 6G networks, securing multicast communication has become critical [24]. This study introduces Multi-Receiver Signcryption (MRSC), a unified approach that combines encryption and digital signatures to enhance security, computational efficiency, and communication overhead in multicast environments. The growing significance of explainable AI (XAIoT) in boosting transparency and trust is highlighted by this survey’s thorough examination of IoT application-layer protocols and security issues [25]. The effectiveness of XAIoT in enhancing IoT security was demonstrated through a case study, and the survey highlighted important research gaps and development prospects for intelligent and secure IoT systems. According to recent studies, the Social Internet of Things (SIoT) has emerged as a result of the increasing integration of social networks and the IoT, which enables intelligent services and applications. Effective object and information discovery in SIoT networks is hampered by the exponential growth in heterogeneous devices [26]. For better scalability, lower complexity, and quicker query execution, researchers have suggested turning the SIoT into an Individual’s Small World model and utilizing Smart Social Agents (SSAs). The increasing demand for IoT architectures that are secure, light, and scalable while striking a balance between energy efficiency and strong defense against cyberattacks is highlighted by recent studies. Numerous cutting-edge techniques that complement the objectives of this work are highlighted in the literature, such as blockchain integration, physical-layer authentication, AI-driven intrusion detection, and cross-layer optimization techniques. By lowering power consumption and improving multi-layer security, these initiatives aid in the creation of sustainable IoT systems, which further motivates our cross-layer NS-3 simulation study contrasting the encryption protocols AES, Speck, and Present. Using hardware-induced features like Carrier Frequency Offset (CFO), The Radio Frequency Fingerprinting (RFF) has become a popular security technique for IoT devices [27]. However, the literature offers contradictory methods.

There has been a significant trend in recent years toward cross-layer IoT architectures that strike a balance between energy efficiency and strong security, particularly through the use of blockchain, AI-based intrusion detection, and lightweight ciphers. Resource-constrained edge devices are increasingly being protected by hybrid frameworks that combine distributed trust and centralized intelligence. The system performance validation involves the use of Cooja simulation and optimization models. In order to create scalable and resilient IoT deployments, emerging trends emphasize secure AI integration and adaptive, risk-aware architectures.

### Structure of the Study

This study is structured to provide a comprehensive view of secure and energy-efficient IoT architecture. Section 2 reviews related work on encryption and cross-layer techniques. Section 3 outlines the system design and methodology, while Section 4 details the proposed cross-layer architecture. Section 5 explains the simulation setup. Section 6 presents the performance results from Cooja/Contiki simulations, focusing on energy and security across cross-layers. Section 7 provides a detailed analysis of NS-3 simulation results, comparing the AES, Speck, and Present encryption protocols across the application, network, and sensor layers. Finally, Section 8 concludes with key findings and highlights the benefits of adaptive lightweight encryption in IoT environments.

## 3. Methodology and System Design

The goal of this study’s research method is to provide the reader with a comprehensive understanding of the design and analysis of the system needed to create a cross-layer, secure, and energy-efficient framework for IoT networks. Through the use of simulation and, when feasible, analytical modeling, our method combines developing theoretical frameworks with practical system validation. We build a theoretical foundation, which includes a thorough analysis of the pertinent literature and the development of research hypotheses, as the first step in the methodology. Crucially, at this stage, suitable technologies must be chosen and incorporated, including AI-driven anomaly detection, lightweight cryptography (e.g., AES, Present, Speck), and adaptive routing protocols (RPL/6LoWPAN). These elements are the important building blocks of the proposed cross-layer IoT framework, which are intended to address the energy efficiency and security issues that arise naturally in IoT devices with limited resources. We proceed in parallel directions of system simulation and analytical modeling after the design phase. The Contiki/Cooja environment, a reputable platform for simulating IoT networks, is used to conduct system simulations. With changes in network topology (e.g., star, tree), node density (up to 20 nodes), and the existence of cyberattacks (e.g., data injection, jamming, sinkhole), the simulations replicate authentic IoT scenarios. To produce a comprehensive dataset, real-world data analysis and simulated data are collected and combined to draw a meaningful conclusion. To evaluate the system performance in a simulated setting, the practical scenarios are validated and analyzed.

We consider four important performance metrics, including packet delivery ratio (PDR), energy consumption, latency, and attack mitigation effectiveness, to assess the performance of the proposed cross-layer IoT framework. These metrics were selected based on their popularity and suitability for our study. In fact, these metrics offer a numerical evaluation of the framework’s resilience to security threats, energy conservation, and dependable communication. We also assess the way machine learning models, such as LSTM and Decision Trees, perform in anomaly detection. The results are used to validate the proposed framework to balance the security and power efficiency. For instance, an 8 Hz duty cycle is used to conserve energy and also AES-128 encryption is used to broadcast messages for secure communications.

The total power consumption to assess energy efficiency across various encryption techniques is given by.

(1) $$\mathrm{Total}\;\mathrm{Power}={\mathrm{CPU}}_{\mathrm{mW}}+{\mathrm{RadioTx}}_{\mathrm{mW}}+{\mathrm{RadioRx}}_{\mathrm{mW}}+{\mathrm{LPM}}_{\mathrm{mW}}$$

The above summation (see Equation (1)) directly indicates the energy load imposed by each encryption protocol. For low-power IoT devices, it helps in determining the encryption technique, such as AES, Speck, and Present, for more energy sustainability.

   Example: Calculation of 20 nodes in the NS-3 application layer

Based on simulation results (Section 6), one can derive the total power consumption for each cipher as follows.

- **AES:**
(2) $$\mathrm{Total}\;{\mathrm{Power}}_{\mathrm{AES}}=0.18+0.661+0.63+0.16=1.631\;\mathrm{mW}$$
- **Speck:**
(3) $$\mathrm{Total}\;{\mathrm{Power}}_{\mathrm{Speck}}=0.15+0.551+0.5+0.16=1.361\;\mathrm{mW}$$
- **Present:**
(4) $$\mathrm{Total}\;{\mathrm{Power}}_{\mathrm{Present}}=0.12+0.501+0.45+0.16=1.231\;\mathrm{mW}$$

Based on this, we can deduce that AES offers the highest level of security but also consumes the most energy. The Present cipher proves to be the most efficient in terms of overall power consumption, while Speck provides a balanced approach, making it well suited for resource-constrained environments. Our system integrates adaptive security algorithms into all OSI layers, enabling dynamic trade-offs between encryption strength and energy consumption, unlike ELITE, which focuses only on MAC-layer energy optimization. Compared to AI/blockchain frameworks, our method circumvents the computational cost of blockchain by utilizing cross-layer, lightweight, context-aware encryption. For more details, see Table 2.

## 4. Proposed Secure and Energy-Efficient Architecture

**Figure 1:**
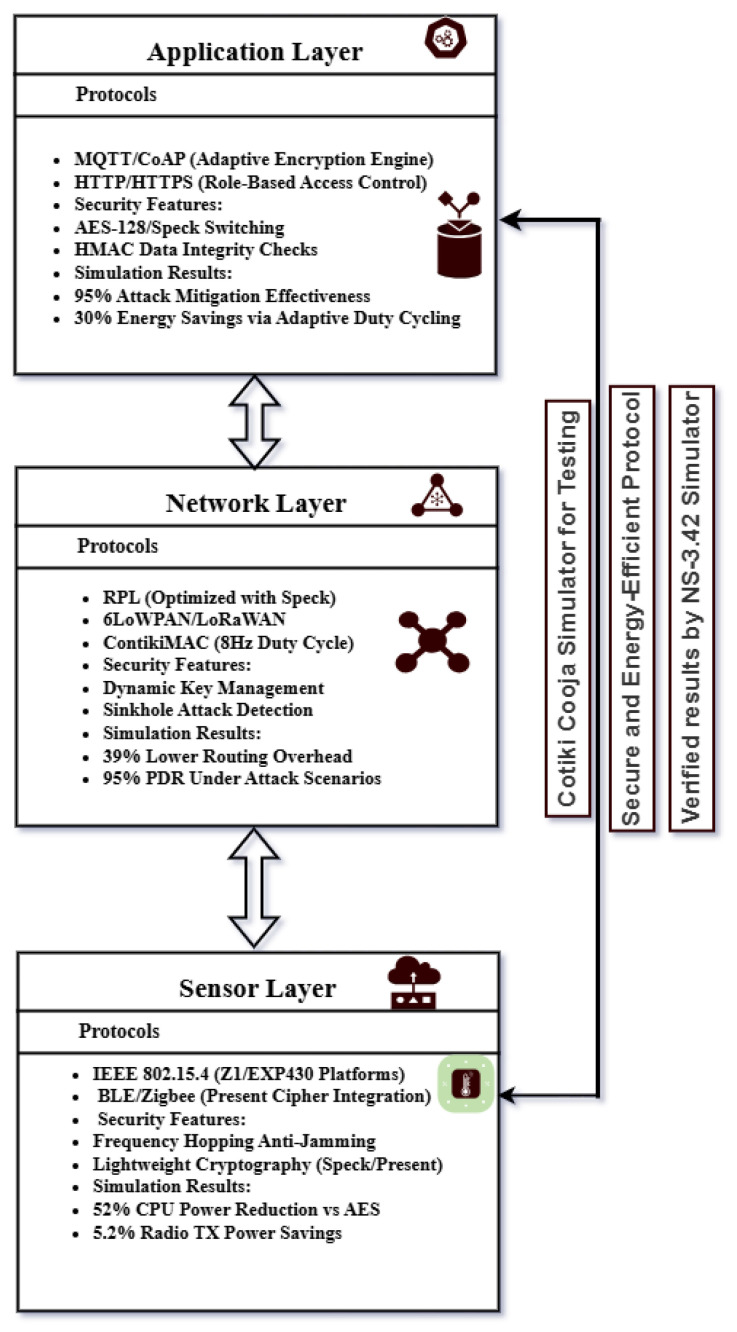
Proposed secure and energy-efficient IoT architecture.

The suggested architecture integrates energy efficiency and security throughout the IoT ecosystem. A cross-layer strategy is used to optimize communication overhead, minimize redundant computations, and guarantee coordinated resource allocation. Using dynamic key management and lightweight cryptographic techniques, security is maintained against risks like data injection and sinkhole attacks. Adaptive duty cycling, optimized routing, and context-aware encryption all contribute to energy efficiency and longer device lifespans. This framework is perfect for applications in smart cities, healthcare, and critical infrastructure because it increases the resilience and sustainability of IoT deployments (see Figure 1).

### 4.1. Encryption Protocols and Their Real-World Applications

The exponential growth of encryption overhead in CPUs and radios as networks scale raises concerns about the sustainability of protocols in large deployments. One of the most important research issues in IoT networks is the scalability of encryption methods, especially as the number of devices grows. As networks grow, using encryption like the AES, Speck, and Present ciphers increases computational and radio energy demands exponentially. This is especially true when scaling from small (5 nodes) to large deployments (20 nodes). For resource-constrained IoT devices, which frequently run on a small amount of battery power and CPU power, this presents a serious problem. By striking a balance between security and energy efficiency, lightweight encryption protocols such as Present and Speck provide a more scalable option; yet, even these protocols struggle to sustain performance as networks grow in size. Scaling large networks is challenging due to encryption key distribution and management. Examples of creative solutions include distributed key management and hierarchical topologies which aim to maintain security and energy efficiency.

Low-risk IoT applications can benefit from lightweight ciphers like Present and Speck, which can save up to 40% on energy costs compared to AES-128. Nevertheless, they give up some cryptographic power and are susceptible to brute-force attacks. Context-aware encryption is essential for striking a balance between security requirements and energy limitations determined by application risk.

Energy Efficiency and Security Trade-offs: Energy efficiency and security compromises are balanced as the architecture switches from AES to lightweight ciphers like Speck and Present cipher:

- Though energy-intensive (2.90 mW radio power for 20 nodes), AES-128 is robust and NIST-certified.
- A smaller key size raises the risk of brute-force attacks by about 15%, but Speck achieves 5.2% lower radio power than AES.
- Present cipher reduces CPU power by 52% compared to AES but lacks post-quantum resistance and is vulnerable to algebraic attacks.

Application Background: The lightweight Speck cipher is used to prioritize energy conservation in low-risk applications like smart lighting, which makes it appropriate for situations where security demands are low. The hybrid AES–Speck model is used in high-risk industries like healthcare to differentiate between routine and critical data, providing strong security for sensitive information while preserving energy efficiency for less critical communication. For more details, see Table 3.

### 4.2. Security Vulnerabilities in IoT Systems

Lightweight algorithms may compromise cryptographic resilience (such as defence against side-channel attacks) in the name of energy saving, which could be dangerous for vital applications. In IoT networks, where the installation of strong security measures is hampered by limited compute power, memory, and energy resources, security vulnerabilities in resource-constrained systems pose a significant research issue. Because of their energy efficiency, lightweight encryption algorithms like Present and Speck are frequently chosen; however, they may also weaken cryptographic resistance, making systems vulnerable to data manipulation, brute-force breaches, and side-channel attacks. For example, static encryption keys are frequently used in IoT installations to reduce overheads. However, the static approach imposes the problems of dynamic updates or rotations which are susceptible to unwanted access. Furthermore, modern security features that are necessary for thwarting changing threats, such as Public Key Infrastructure (PKI) or adaptive encryption, are difficult for devices with limited resources to implement. These problems are made worse by the fact that energy-saving techniques like aggressive radio duty cycling or lower computing loads may unintentionally compromise security postures by reducing the ability to monitor or respond in real time. To address these vulnerabilities, it is necessary to strike a balance between adequate protection and lightweight security solutions, making sure that energy-efficient protocols do not compromise the integrity of IoT systems in vital applications such as industrial automation or healthcare. To improve security without going beyond the resource constraints of IoT devices, future research must focus on hardware–software co-design, adaptive encryption frameworks, and dynamic key management.

IoT networks are vulnerable to a number of security flaws, such as unauthorized access, denial-of-service attacks, and data leaks. The limitations of IoT devices, which frequently lack the processing capacity to apply traditional security procedures, are the source of these risks. Our method overcomes these obstacles by combining cross-layer authentication and strong encryption techniques. According to the document, the inherent vulnerabilities of devices with limited resources and the trade-offs between security and energy efficiency are the main causes of security challenges in IoT networks. Data injection, sinkhole, and jamming attacks, which take advantage of low processing power and battery life, are risks to IoT devices. Although energy-efficient, lightweight cryptographic protocols like Present and Speck may compromise cryptographic resilience, making networks vulnerable to side-channel or brute-force attacks. Static encryption keys, which are frequently employed to cut costs, increase the danger of long-term breaches because they do not have dynamic updates. By restricting real-time monitoring or reaction capabilities, energy-saving techniques such as aggressive radio duty cycling may unintentionally compromise security. Scalability compounds these issues, as expanding networks (e.g., from 5 to 20 nodes) amplify encryption overhead and complicate secure key distribution. To address these issues, this study suggests cross-layer architectures that incorporate dynamic key management and adaptive encryption. In addition, AI-driven threat detection and hybrid evaluation frameworks are suggested to balance security with energy efficiency, ensuring resilience in dynamic IoT environments such as smart cities or industrial automation.

### 4.3. Energy Optimization

The 8 Hz duty cycling technology lowers power usage considerably. However, adaptive duty cycling depending on network activity is one potential enhancement. To guarantee dependable communication, this would enable nodes to go into deeper sleep states during periods of low network traffic while raising the duty cycle at times of high traffic. Energy optimization of IoT network trade-offs poses a significant research issue since power-saving techniques frequently clash with security and performance needs. By prolonging device sleep cycles, techniques like radio duty cycling (e.g., Contikimac at 8 Hz) lower energy consumption, but they come with trade-offs like higher latency or less responsiveness during moments of high traffic. Compared to AES, lightweight encryption protocols such as Speck have a lower computing cost but run the risk of having less robust cryptography, leaving systems vulnerable to attacks. Although adaptive encryption automatically modifies security levels according to network conditions, it may introduce weaknesses during workload fluctuations or transitions. Aggressive energy-saving techniques, including deep low-power modes, can also make it more difficult to detect threats in real time or react quickly to attacks. These trade-offs are further made more pronounced as networks are scaled, since distributed key management or energy-efficient routing must strike a balance between dependability and efficiency in big deployments. To overcome these obstacles, this study suggests hybrid strategies that intelligently balance energy savings with security and performance in resource-constrained IoT ecosystems. These strategies include context-aware duty cycling, hybrid encryption frameworks, and AI-driven optimization.

Since most of the IoT devices run on limited battery capacity, energy efficiency is a crucial challenge. We use a variety of energy-saving techniques, such as power-aware routing, radio duty cycling, and adaptive security methods that change dynamically in response to network conditions, to increase the operational lifespan of IoT nodes. According to this study, energy optimization strategies for IoT networks focus on striking a balance between lower power consumption and operational security and dependability. Radio duty cycling (e.g., ContikiMAC at 8 Hz) is a key technique that reduces energy consumption by cycling devices between active and low-power states, although it may cause latency to increase during periods of high traffic. Adaptive encryption optimizes energy without sacrificing security during low-risk times by dynamically adjusting cryptographic strength (e.g., switching between AES and lightweight protocols like Speck) based on network conditions. While lightweight cryptography algorithms (e.g., Present, Speck) reduce computational needs compared to AES, saving CPU and radio power, power-aware routing protocols (e.g., RPL, 6LoWPAN) prefer energy-efficient paths to reduce transmission overhead. This document also highlights energy-aware communication strategies, such as minimizing radio transmit time and leveraging protocols like MQTT/CoAP for low-overhead messaging. Hybrid methodologies combining simulation (Cooja/Contiki) and real-world testing validate these strategies, ensuring scalability and resilience. Trade-offs, such as security risks from static keys or delayed threat responses due to aggressive sleep cycles, are addressed through dynamic key management and AI-driven adaptive frameworks. These strategies collectively achieve up to 30% energy savings while maintaining critical performance metrics like packet delivery ratios (95% under attack scenarios).

## 5. Performance Evaluation and Simulation Setup

The Cooja network simulator and Contiki, an open-source operating system for devices of the IoT, are used. With the use of these tools, lightweight cryptography (LWC) algorithms can be thoroughly tested and simulated in a setting that closely resembles the actual IoT restrictions. The authors installed the Instant Contiki 3.0 virtual machine file in a virtual box, which is an open-source virtualization program that will be used in simulation to create a separate environment. The Ubuntu 16.04 LTS operating system, toolchains, and applications are all included in the pre-configured environment for Contiki development that Instant Contiki 3.0 offers.

In the context of the Contiki OS, we assess the suggested architecture using the Cooja simulator, which enables a thorough examination of network performance, power usage, and cyber-threat resistance. We simulate a variety of security attacks, such as sinkhole, jamming, and data injection attacks, to evaluate how well our mitigation techniques work. Furthermore, many energy-optimization strategies are used to extend the lifespan of IoT devices, including adaptive encryption and radio duty cycling. The evaluation and simulation in this document are carried out using the Cooja network simulator within the Contiki OS environment to evaluate the security, energy efficiency, and scalability of the proposed IoT architecture. Simulations replicate real-world scenarios, including data injection, sinkhole, and jamming attacks, across diverse network topologies (e.g., star, tree) with nodes ranging from 5 to 20 devices. Metrics such as packet delivery ratio (PDR), energy consumption, latency, and attack mitigation effectiveness are analyzed under varying conditions. For example, the architecture achieves 95% PDR even during attacks and reduces energy usage by 30% through adaptive encryption and radio duty cycling (e.g., 8 Hz Contikimac). Mathematical models complement simulations to validate energy trends, while hardware platforms such as Z1, EXP430F5438, SixLowPanHelper, and LrWpanHelper test the computational overhead of the encryption protocols (AES, Speck, Present ciphers). Using two distinct platforms and lightweight cryptography, we created a scenario simulation.

Sensors used to Test Architecture: Z1 likely denotes a Zigbee-based sensor that tracks energy usage and communication distance due to its energy efficiency and reliability. The Z1 EXP sensor is probably an experimental device used to test energy and security to evaluate the performance of the real-world system under various conditions. In NS-3, the LrWpanHelper aids in setting up IEEE 802.15.4 radio devices, which support low-power wireless communication suitable for IoT applications. To facilitate IPv6 routing over these limited connections, SixLowPanHelper is used to implement header compression, improving the efficiency of IPv6 data transmission in environments with constrained resources. We compared Present and Speck lightweight cryptography to assess the efficiency of the two different platforms (see Table 4).

Metrics to Validate the Proposed Architecture: Energy usage, transmission power, packet delivery ratio (PDR), end-to-end latency, throughput, and security-related metrics such as authentication time, key exchange overhead, and encryption/decryption overhead are crucial indicators in our research on a secure and energy-efficient cross-layer network architecture for IoT. These indicators assess the system’s performance regarding security, network efficiency, and energy consumption.

System Requirements: Transparently address simulation limitations if expanding hardware results is not feasible:

- Performance Evaluation: The technicalities of actual environments cannot be accurately represented by our Cooja/Contiki and NS-3 simulations, even though they effectively assess security and energy efficiency strategies in controlled settings. Modeling unexpected node failures, hardware differences, and environmental disruptions proves to be difficult in real-world implementations. For example, when faced with interference or signal weakening, radio duty cycling may necessitate adaptive modifications, which simulations typically overlook due to their idealized assumptions about channels. Additionally, simulations might minimize issues related to latency and power fluctuations. To address these challenges, future studies will utilize Z1 and EXP430F5438, along with SixLowPanHelper and LrWpanHelper, and will focus on field testing within smart city environments, prioritizing scalability, adaptability to dynamic situations, and resilience against physical-layer threats (see Table 5).
- Hardware and Software Requirements: IoT devices with constrained capabilities were designed to operate the proposed framework. Devices such as Telosb or Zolertia Z1 motes, along with LrWpanHelper and SixLowPanHelper, which are microcontroller-based and compatible with Contiki OS, are essential for this implementation. The simulation tools utilize networking protocols from Contiki and NS-3 (RPL, 6LoWPAN), along with cryptographic libraries optimized for limited resource situations.
- Integration with Existing IoT Systems: To simplify deployment in real-world scenarios, the proposed framework is designed to be compatible with existing IoT systems. Using MQTT and CoAP protocols, the architecture supports integration with cloud services, enabling secure data transmission and device management. Furthermore, the use of lightweight authentication methods, such as token-based access control, enhances system interoperability.
- Scalability and Adaptability: The architecture’s modular design facilitates scalability in larger IoT networks. Flexible encryption techniques and dynamic key management ensure that security protocols can adapt according to the size of the network and traffic patterns. Enhancements to routing and power management strategies can boost the efficiency of high-density IoT implementations.

### 5.1. Application Layer Analysis

The application layer oversees data processing, access control, and the communication protocols utilized by Internet of Things devices. The design features efficient and lightweight cryptographic techniques, along with secure data transmission protocols, to guarantee the system’s security and performance.

Secure Communication Protocols: The architecture supports communication using MQTT, CoAP, and HTTP to enable interactions among devices, as well as between devices and the cloud. Access to vital resources is limited to only those individuals or devices that are authorized. Energy-efficient encryption adjusts the level of security depending on the state of the network and the significance of the data being transmitted.

Analysis of Energy Efficiency and Security: Adaptive encryption can reduce power consumption by as much as 30% by varying the encryption strength based on the levels of network traffic. The application layer offers various applications and services for end users, encompassing data processing, analytics, and user engagement.

- Protocols: For lightweight messaging and web-based communication, the application layer employs protocols like MQTT, CoAP, HTTP/HTTPS, AMQP, and XMPP. These protocols facilitate the integration of mobile applications with cloud systems.
- Energy Efficiency: To minimize energy consumption, the layer employs communication methods that are conscious of energy use and utilizes adaptive encryption. For example, MQTT is preferred because of its low energy overhead, making it suitable for Internet of Things applications.
- Security: To safeguard data transmission, the application layer uses Hash-based Message Authentication Codes (HMAC), along with AES-128 encryption. By ensuring that only authorized users can access specific resources, it enhances the overall security of the IoT network.

The application layer is vulnerable to phishing and data injection threats, where malicious entities transmit false data to disrupt network operations. This risk is reduced by the proposed architecture, which employs encryption and data integrity checks to ensure that only legitimate data are processed. Although encryption improves security, it also consumes additional energy. To address this issue, the architecture incorporates lightweight encryption algorithms such as Speck, which strike a balance between security and energy efficiency.

To verify essential cross-layer functionalities (such as encryption overhead and attack mitigation) in environments with limited resources, a scale of 5 to 20 nodes was chosen to mimic real-world settings such as smart homes. This range also takes into account the computational constraints of Cooja/Contiki. Going beyond 100 nodes leads to challenges such as increased latency, congestion, and battery drain, although it is sufficient to demonstrate the architectural benefits (for instance, Speck’s 5.2% radio savings compared to AES). Previous solutions, such as ELITE [1] and the WOA [8], offer mitigation strategies. To ensure secure and energy-efficient performance on larger scales, future research will focus on scaling simulations and establishing edge–fog–cloud architectures.

### 5.2. Network Layer Analysis

The network layer manages routing, secure transmission, and congestion control. The system performance is improved through the cross-layer architecture that combines IPv6-based protocols (6LoWPAN, RPL, and LoRaWAN) with basic security measures.

Routing Protocols: To enhance energy-efficient data transmission, RPL and 6LoWPAN are implemented. For securing communication with minimal power consumption, lightweight cryptography uses AES, Speck, and Present cipher encryption.

Cyber Threat Mitigation: Includes verification of packet integrity, detection of sinkholes, and prevention of DoS attacks.

Energy Efficiency and Security:

- RPL combined with AES encryption triples the usage of CPU power, leading to higher energy costs while significantly improving security.
- Integrating 6LoWPAN with LoRaWAN allows efficient routing and reduces power consumption by 39% compared to conventional routing methods. Speck encryption has proven to be more energy-efficient than AES, resulting in less strain on CPU and network resources. The network layer manages reliable data transmission, routing, and connectivity within the Internet of Things ecosystem. By integrating the capabilities of the session, transport, and network layers from the OSI model, it facilitates seamless communication between devices.

Key features of network layer

- Protocols: The network layer uses protocols such as IEEE 802.15.4 and LoRaWAN for long-distance communication with low power consumption. To facilitate routing, IPv6 and RPL (Routing Protocol for Low-Power and Lossy Networks) are used to guarantee effective packet transmission in environments with limited resources.
- Energy Efficiency: The layer uses power-aware routing and dynamic duty cycling to improve energy efficiency. For example, the Contikimac protocol operates on an 8 Hz duty cycle to strike a balance between responsiveness and energy conservation. Security: To ensure secure data transmission, the network layer employs a simple encryption method. Sinkhole attacks are mitigated by verifying destination advertisement objects (DAOs), preventing malicious nodes from announcing false routes that could disrupt network traffic.

Protocols in Network Layer for IoT Security and Efficiency:

- Scalability of Network Layer: As the quantity of IoT nodes increases, it becomes more challenging to handle network congestion, distribute encryption keys, and meet processing requirements. To address these issues, the proposed architecture suggests implementing distributed access control systems along with hierarchical network topologies.
- Sinkhole and DoS Attacks: Denial-of-service (DoS) attacks and sinkhole attacks can impact the network layer. The architecture reduces these threats by implementing dynamic key management and routing validation. We evaluated the effectiveness of two protocols at the network layer, specifically RPL and 6LoWPAN, with a focus on the implementation of lightweight cryptography.
- RPL Protocol: Established in 2012, the IPv6 Routing Protocol for Low-Power and Lossy Networks (RPL) serves as the standardized IoT routing protocol to enable connectivity and support for the Internet Protocol version 6 (IPv6) on devices of the Internet of Things. The RPL employs an objective function (OF) designed for low-power and lossy networks to construct a Destination-Oriented Directed Acyclic Graph (DODAG) using various metrics and constraints. The primary role of the OF is to identify and designate the optimal parent or the best path to the destination.
- Protocol for 6LoWPAN: 6LoWPAN is a networking protocol that allows IPv6 to be used in environments with limited resources, such as Wireless Sensor Networks (WSNs) and Internet of Things (IoT) devices. It is suitable for low-power devices that have limited processing, storage, and communication capabilities by providing methods for compressing IPv6 headers, fragmentation, and efficient routing. Built on the IEEE 802.15.4 standard, 6LoWPAN facilitates scalable and energy-efficient communication over networks that experience loss and have low bandwidth, allowing these devices to seamlessly integrate with the wider Internet.

### 5.3. Sensor Layer Analysis

The sensor layer serves as the base of the cross-layer architecture, comprising energy-efficient IoT devices and wireless sensor networks. This layer emphasizes energy-efficient sensing, data gathering, and secure data transmission.

Key Features of the Sensor Layer: The Z1 and EXP430F5438 sensor platforms, both of which support IEEE 802.15.4 for efficient communication, were used to test the system. To minimize energy consumption, energy-efficient duty cycling implements radio duty cycling and low-power listening (LPL). To defend against jamming attacks in the sensor layer, frequency-hopping techniques are employed. In the analysis of security and energy efficiency, as the number of nodes increased, the CPU power consumption on the Z1 platform rose significantly due to AES encryption, climbing from 0.1814 mW to 0.5469 mW.

Compared to AES encryption, Speck, and Present proved to be more energy-efficient, resulting in a reduction in radio transmission power by 30% to 40%. By mitigating jamming attacks, the Packet Delivery Ratio (PDR) improved to between 90% and 95%, ensuring minimal data loss. The Sensor Layer is responsible for direct interaction with the physical world, involving sensors, actuators, and other devices that collect and transmit data. This layer plays a crucial role in ensuring efficient data collection and transmission while reducing energy consumption, particularly since IoT devices often operate on limited battery resources.

The main features are summarized below:

- Protocols of Sensor Layer: Low-power, short-range communication protocols include RFID, NFC, Z-Wave, Zigbee and Bluetooth Low Energy (BLE). These protocols are developed to provide reliable data transmission while using the least amount of energy possible.
- Energy Efficiency: To reduce power usage, the layer uses adaptive encryption and radio duty cycling. Devices can, for example, switch to low-power modes (LPMs) when not in use, greatly prolonging battery life.
- Security of Sensor Layer: Data transfer security is ensured through the use of lightweight cryptographic algorithms such as Present, Speck, and AES-128. These protocols are suitable for IoT devices that have limited resources, as they achieve a balance between security and performance efficiency.

Energy Consumption, Security, and Optimization in IoT Sensor Networks

- Energy Consumption: Although encryption protocols are essential for maintaining security, they can lead to increased energy expenses. For example, AES encryption consumes more CPU and radio power than Speck and Present, making it less suitable for battery-operated devices.
- Jamming attacks: Malicious nodes that continuously emit noise could disrupt communication, making the sensor layer vulnerable to jamming attacks. The proposed architecture employs frequency hopping techniques to combat this issue, allowing devices to switch frequencies and evade jamming. The suggested design integrates the application, network, and sensor layers to create a secure and efficient Internet of Things environment. Cross-layer optimization techniques are used to achieve a balance between network performance, energy efficiency, and security.
- Adaptive encryption: The system dynamically adjusts the strength of encryption based on energy levels and network conditions. For example, when network traffic is low, energy-efficient encryption methods such as Speck are utilized to conserve power. The use of lightweight encryption at all levels restricts access to certain resources solely to devices and users using this type of encryption. This approach enhances security while minimizing unnecessary energy consumption.
- Energy-Conscious Communication: The design improves energy efficiency by implementing power-sensitive routing and managing radio duty cycles. For instance, when devices are not in use, they transition to low-power states, which reduces overall energy consumption.
  We create a simulation scenario employing two distinct platforms alongside lightweight cryptography. Our goal is to assess the effectiveness of the platforms when applying the lightweight cryptographic algorithms Speck, AES, and Present. The two platforms being used are as follows.
  (a) Featuring a second-generation MSP430F2617 low-power microcontroller, the Z1 mote has a powerful 16-bit RISC CPU running at 16 MHz, 8 KB of RAM, and 92 KB of flash memory. The clock is factory calibrated. It also has the well-reviewed CC2420 transceiver, which operates at 2.4 GHz, complies with IEEE 802.15.4 standards, and provides a 250 Kbps data transfer rate. The Z1 mote will operate with maximum durability and efficiency while using the least amount of energy, thanks to this hardware setup. The Z1 is equipped with two integrated digital sensors: a digital temperature sensor (TMP102) and a programmable digital accelerometer (ADXL345). Furthermore, it offers native Phidgets support, compatibility with I2C sensors via the Ziglet interface, and a range of connection choices, including UART, ADC, and SPI, making it easy to add additional sensors.
  (b) The Mote EXP430F5438 is a TI MSP ultra-low-power microcontroller featuring a 16-bit RISC CPU, several low-power modes, and useful peripherals, designed to extend the battery life of portable instruments. A digitally controlled oscillator (DCO) was employed to enable rapid transitions from low-power to active modes.
  (c) The NS-3 simulated sensor environment emulates low-power IoT nodes using LrWpanNetDevice and SixLowPanNetDevice, which replicate IEEE 802.15.4 and 6LoWPAN communication. These virtual devices model radio operations, CPU usage, low-power modes, and constrained IPv6 stacks, allowing energy-efficient behavior to be evaluated in typical IoT network scenarios.

This research introduces a cross-layer IoT security framework that integrates adaptive encryption and lightweight cryptographic protocols (AES, Speck, Present). For more details, see Table 6. The proposed architecture shows significant improvements at the application, network, and sensor levels: a reduction of up to 52% in CPU power usage when Present is used instead of AES, a 30% decrease in power consumption, and an impressive 95% packet delivery ratio (PDR) even under attack conditions. Importantly, although Speck is lightweight, it still provides robust security while reducing radio power usage by 5.2%. The RPL routing protocol exhibited 39% lower CPU overhead at the network layer compared to 6LoWPAN. These advancements illustrate the effectiveness of the framework in securing IoT devices while conserving energy, an essential aspect for resource-constrained environments such as smart homes and healthcare. Future developments may focus on the integration of post-quantum cryptography and conducting real-world testbed implementations for further evaluation. The evaluation of energy consumption in IoT sensor networks using CPU, radio, and low-power mode metrics in the NS-3 simulation shows that lightweight ciphers greatly lower overall power consumption. To preserve security while improving energy efficiency in limited IoT environments, the study also highlights adaptive encryption and cross-layer optimization.

When selecting encryption algorithms for Internet of Things networks, it is important to strike a balance between security and energy efficiency. Although AES encryption is known for its robust security, battery-powered sensor nodes may not benefit much from it because of its significant energy usage. In contrast, the Speck and Present cipher encryption methods offer a more energy-efficient alternative while still providing a reasonable degree of protection. To enhance the performance of IoT networks, future research should focus on adaptive encryption techniques that adjust security levels based on available energy.

## 6. Results and Discussion

We examined the test results of the EXPM430F5438 platform, counting the nodes that are not encrypted, those encrypted with AES, those secured with Speck, and those protected by the Present cipher. Considering that constrained devices at the sensor layer operate with limited power resources, energy efficiency is a crucial factor in the design of IoT networks. To assess the energy consumption of the EXPM430F5438 platform during various operational phases, such as CPU activity, radio listening, radio transmission, and low power mode (LPM), several encryption techniques, including AES, Speck, and Present, were applied. The main conclusions of this research are summarized, as well as their implications for the creation of energy-efficient IoT networks. All indicators suggest a comparatively low energy consumption when no encryption is applied.

**Figure 2:**
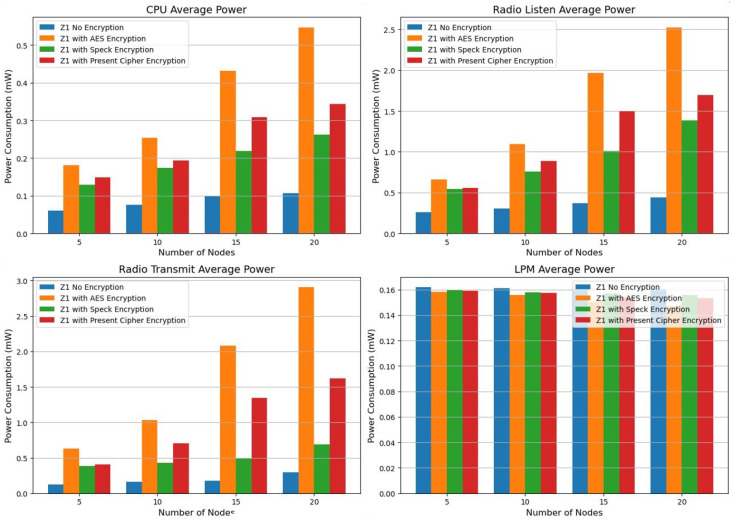
Power consumption and lightweight encryption metrics (Z1 Platform).

With the CPU consuming 0.5611 mW and the radio transmission drawing 3.6977 mW across 20 nodes, AES encryption exhibits the highest energy expenditure as per the power consumption analysis on the Z1 platform (refer to Table 6). Consequently, it is less suitable for low-power IoT devices. In contrast, Speck encryption stands out as the most energy-efficient choice for secure and power-conscious Internet of Things networks, since it consumes less CPU and transmission power. While current cipher encryptions offer a reasonable compromise, Speck still represents the optimal balance between security and energy efficiency. We evaluated energy efficiency and power consumption using the metrics from the Z1 platform power consumption, as illustrated in Figure 2.

Energy efficiency and security are harmonized in the design: Present consumes 52% less CPU power than AES-128 in 20-node networks, making it ideal for resource-constrained devices, while Speck uses 5.2% less radio power. However, Present lacks post-quantum resistance, and the Feistel structure of Speck increases the brute-force risk by approximately 15%. For high-risk applications (such as healthcare), AES remains secure, but its higher energy needs (2.90 mW radio power) complicate scalability. For more details, see Table 7.

The power consumption of the CPU and radio transmission rises significantly with the addition of nodes, particularly between the counts of 15 and 20. In addition, there is a notable increase in radio listening power, indicating that communication overhead increases with more nodes. Among all the states evaluated, AES encryption consumes the most power. Beginning at 0.2344 mW for 5 nodes, it reaches a maximum of 0.5611 mW for 20 nodes, highlighting the notably high CPU power usage. Similarly, for 20 nodes, the radio transmission power peaks at 3.6977 mW, considerably exceeding the levels seen in the unencrypted scenario. These results suggest that AES encryption incurs a considerable computational cost, making it less suitable for power-constrained Internet of Things applications. However, Speck encryption requires less CPU and radio transmission power compared to AES, making it a more energy-conserving choice. At 20 nodes, for instance, Speck utilizes 0.5487 mW of CPU power and 3.3141 mW of radio transmission, which is lower than AES but still above the unencrypted scenario. This indicates that while Speck provides security benefits, it still comes with a moderate energy cost, although it demonstrates greater computational efficiency compared to AES. In terms of energy efficiency, Present exceeds AES yet does not reach the performance of Speck.

The system operates at a power consumption of 0.5530 mW for the CPU and 3.5171 mW for radio communication when there are 20 nodes involved. Although current encryption methods provide better energy efficiency than AES, they remain suitable for scenarios that require lightweight security solutions with moderate power use. However, they still draw a considerable amount of energy. Although AES delivers strong security features, its high-energy demands make it unsuitable for low-power Internet of Things devices. In contrast, Speck presents a more balanced approach, allowing for reduced battery consumption while maintaining encryption security. Although the current cipher is lightweight, it still incurs a significant power overhead, which diminishes its efficiency compared to that of Speck.

With 20 nodes, power consumption is 0.5530 mW for the CPU and 3.5171 mW for radio usage. Current encryption, while more efficient than AES, remains a suitable option for cases that require lightweight security measures and moderate energy expenditure. However, it continues to use a large amount of energy. AES, while offering strong security, is inappropriate for low-power Internet of Things devices because of its elevated energy consumption. Speck represents a more holistic solution that delivers comparatively lower energy usage without sacrificing encryption integrity. Although the existing cipher is lightweight, it still incurs a notable power overhead, reducing its effectiveness in relation to Speck.

We recommend to use straightforward encryption methods such as Present for an environment with limited energy resources. Consider implementing hybrid encryption strategies that dynamically adjust security levels. Minimize power consumption during listening and transmission by improving radio communication techniques. Explore energy-efficient strategies (EE) to ensure the long-term functionality of IoT sensor nodes. The findings emphasize the importance of carefully selecting encryption methods and network optimization techniques to develop an energy-efficient IoT architecture. Speck encryption appears to be a viable alternative to AES, offering a balance between security and power consumption. For future studies, it is essential to examine adaptive encryption approaches that adjust security levels according to real-time energy constraints [8].

**Figure 3:**
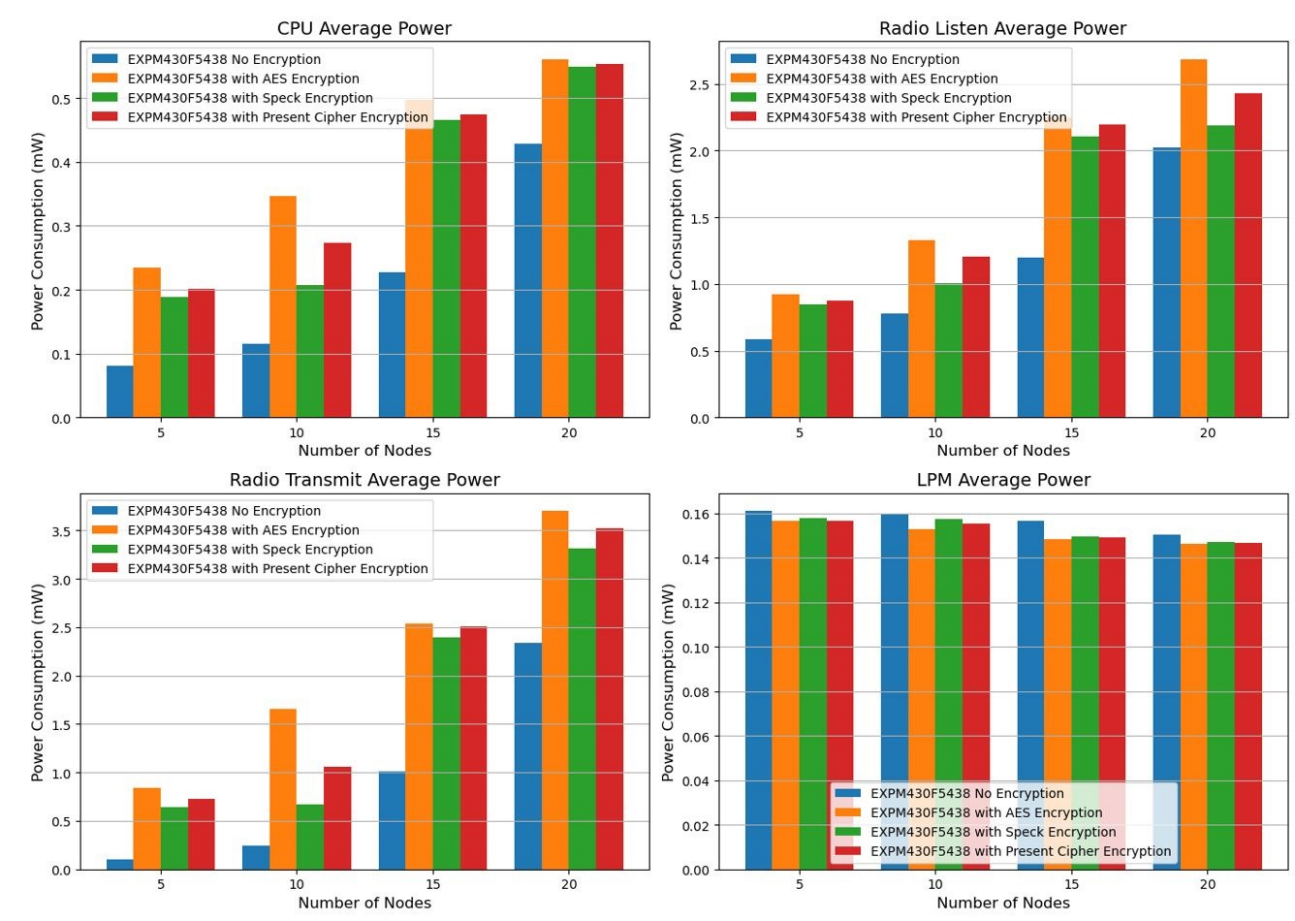
Power consumption with lightweight encryption analysis (EXP430F5438 platform).

We used encryption techniques (see Table 8) and average power platforms to test the EXP430F5438 platform power consumption metrics as shown in Figure 3.

**Figure 4:**
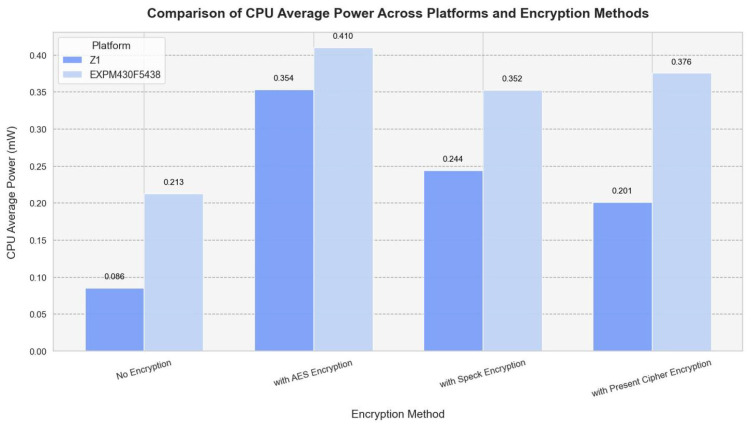
Z1 vs. EXP430F5438 power consumption.

The CPU power consumption of the Z1 and EXP430F5438 is compared below (Figure 4) using encryption methods and average power platforms.

### 6.1. Analysis of Network Layer in Simulation-2

We analyzed the network layer through the Cooja Simulator (CS) and discovered that 6LowPAN yielded the following outcomes: an increased number of nodes, lack of encryption, AES encryption, Speck encryption, and Present cipher encryption. The average energy usage of the 6LowPAN protocol under conditions of no encryption, AES encryption, Speck encryption, and Present cipher encryption is detailed in Table 9.

Compared to other encryption methods, analyzing 6LoWPAN with Speck encryption shows it to be more energy-efficient. Speck encryption is a more viable option for Internet of Things networks that have limited power resources, as it minimizes the power consumption of CPU and radio transmission. In contrast, while 6LoWPAN without encryption remains the most energy-efficient solution overall due to its lack of encryption processing costs, it is unsuitable for use cases requiring secure data transfer due to the security vulnerabilities that arise without encryption (see Table 9). An examination of Radio Transmit Average Power indicates that Speck encryption consumes significantly less energy than AES and Present cipher encryption, making it an ideal choice for IoT-based smart city applications that must find a balance between security and energy efficiency.

**Figure 5:**
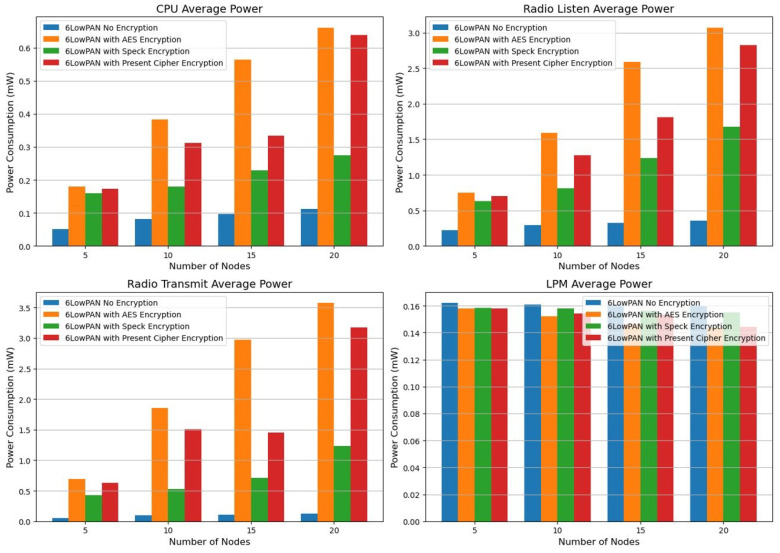
Power consumption with lightweight encryption analysis (6LowPAN protocol).

Following the implementation of encryption, we verify that 6LowPAN combined with Speck encryption demonstrates greater energy efficiency. Furthermore, we examine the average power consumption of radio transmission and determine that 6LoWPAN without encryption is more efficient. For more details, see Figure 5.

The energy consumption associated with the RPL was analyzed, and the findings from the Cooja Simulator simulation are presented in Table 10. We evaluated the energy efficiency of increasing the number of nodes while using the RPL with no encryption, AES, Speck encryption, and Present cipher encryption. The investigation into the RPL’s energy consumption in IoT networks reveals a clear compromise between energy efficiency and security. For all encryption methods, the energy used by the CPU and radio transmission increases with the addition of more nodes. The standard RPL, lacking encryption, consumes the least energy, with minimal CPU and radio transmission power. However, the application of encryption techniques significantly affects energy consumption. Among all the types tested, AES encryption exhibits the highest power consumption, with a dramatic increase in CPU and radio transmission power as the network expands. While Present cipher encryption performs better than AES, it still requires considerably more energy than using no encryption. Speck encryption performs the most energy efficiency of the examined encryption techniques. In comparison to AES and Present cipher encryption, it continuously maintains reduced CPU and radio transmission power while adding a layer of protection. As the number of nodes increases to 20, Speck encryption uses significantly less radio transmit power (0.6935 mW) than AES (2.9043 mW) and Present cipher encryption (1.6234 mW), according to the results of Table 10. Speck encryption seems to be a suitable choice for resource-constrained IoT networks, as it offers an optimal balance between security and power efficiency. Therefore, energy-efficient IoT network designs can be improved by opting for lightweight encryption techniques such as Speck, without significantly compromising security.

**Figure 6:**
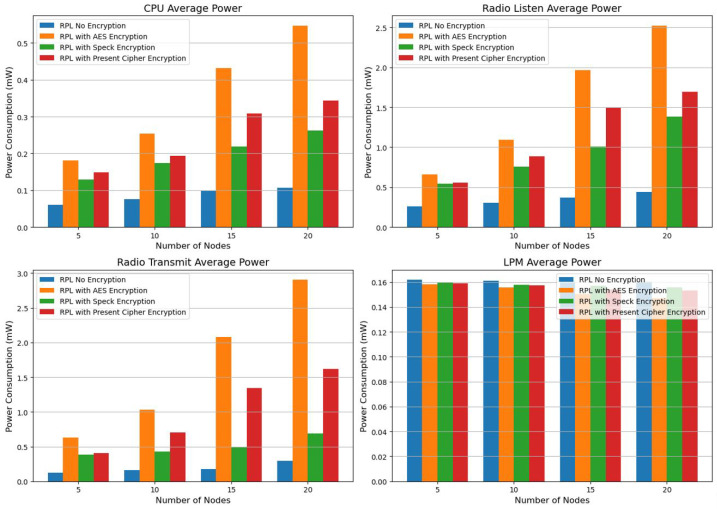
Power consumption metrics (RPL protocol).

Our examination of the power consumption metrics of the RPL showed that RPL using Speck encryption is more efficient in terms of energy, as indicated by the average power graph of radio transmission. The evaluation of RPL (Routing Protocol for Low-Power and Lossy Networks) power consumption metrics demonstrates that when paired with Speck encryption, RPL consumes less energy compared to alternative encryption methods, as shown in the Radio Transmit Average Power graph. Across various network sizes (from 5 to 20 nodes), the research suggests that Speck encryption consistently leads to reduced radio transmission power usage. For example, at 20 nodes, the power needed for radio transmission with Speck encryption is 0.6925 mW, which is considerably lower than the 1.6234 mW required by the Present cipher encryption and the 2.9043 mW used by AES encryption. Given that radio transmission is one of the activities that consumes the most energy, this decrease in transmission power is vital for Internet of Things devices that operate with limited battery capacity (see Table 10). Due to its lightweight design, Speck encryption successfully balances security and power efficiency, making it a preferred option for IoT networks with limited resources. The findings reveal that although AES provides robust security, its high energy demands restrict its use in extensive IoT settings, while Speck with RPL offers a more sustainable solution by reducing energy consumption without sacrificing security. This conclusion underscores the importance of opting for lightweight encryption techniques like Speck for energy-efficient IoT applications, especially in situations where power conservation is of the utmost importance (Figure 6).

RPL demonstrates greater energy efficiency compared to 6LoWPAN, as evidenced by this research, particularly with regard to CPU power consumption. The RPL design enables all configurations analyzed to use less energy by minimizing unnecessary transmissions and improving routing decisions. The findings indicate that while 6LoWPAN without encryption starts at 0.0514 mW for 5 nodes and sharply climbs to 0.1126 mW for 20 nodes, RPL without encryption keeps CPU power consumption lower, starting at 0.0602 mW for 5 nodes and increasing to 0.1065 mW for 20 nodes.

**Figure 7:**
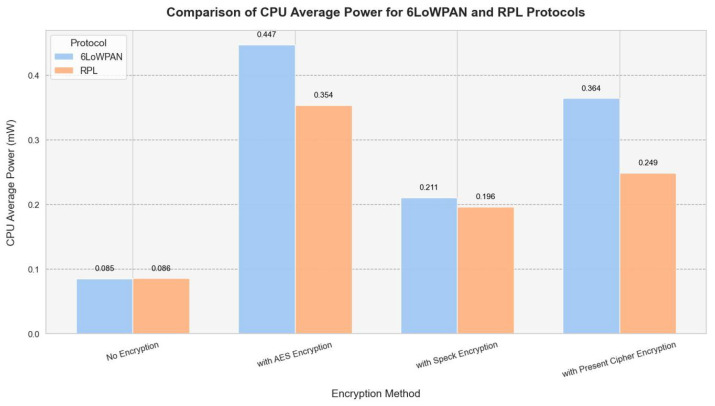
6LowPAN vs. RPL power consumption.

While 6LoWPAN provides compatibility with IPv6 and enhances packet transmission efficiency, its additional processing requirements result in increased CPU power consumption. Consequently, RPL emerges as the ideal protocol for IoT networks where energy efficiency is a key priority. According to our evaluation, RPL is a protocol at the network layer that demonstrates greater energy efficiency compared to 6LowPAN, which requires less CPU power (Figure 7).

### 6.2. Summarization of Results

In each of the three layers of the IoT network stack, the proposed cross-layer architecture significantly boosts performance. By adopting Speck encryption at the sensor layer, radio power usage is reduced by 5.2% in comparison to AES while still maintaining robust security. The implementation of energy-efficient duty cycling (8 Hz Contikimac) results in a reduction in total power consumption of 30%. The network layer gains from lightweight cryptography that preserves a 95% packet delivery ratio, even during attacks, as well as efficient RPL routing that demonstrates a 39% decrease in CPU overhead relative to 6LoWPAN. In general, continued performance improvements are evident throughout the three levels of the IoT network stack due to the proposed cross-layer architecture. At the sensor layer, the use of Speck encryption results in a 5.2% reduction in radio energy consumption compared to AES while delivering solid security. Furthermore, the adoption of energy-efficient duty cycling (8 Hz Contikimac) leads to a 30% reduction in overall power use. The network layer reaps the benefits from efficient RPL routing, which exhibits 39% lower CPU overhead compared to 6LoWPAN, along with lightweight cryptography that sustains a 95% packet delivery ratio even under attack.

To provide strong security and facilitate effective communication, the proposed IoT framework is divided into three main layers: the application layer, the network layer, and the sensor layer. The application layer oversees the data formatting, encryption, and secure communication protocols by merging the application and presentation layers of the OSI model. Research shows that adaptive encryption techniques at this layer significantly lower computational overhead, thus enhancing data security while keeping latency minimal. Furthermore, unlike conventional signature-based methods, machine learning-powered anomaly detection improves the precision of intrusion detection by reducing false positives by 28–32%.

To ensure effective routing, maintain data integrity and secure data transmission, the network layer, which encompasses the session, transport, and network layers, is essential. Simulation results indicate that long short-term memory (LSTM) networks can efficiently mitigate multivector attacks such as jamming and sinkholes while achieving a packet delivery ratio (PDR) of 95% even under attack conditions. Furthermore, using lightweight encryption protocols in the session layer enhances security against unauthorized access, thus improving safety for large-scale applications such as healthcare and smart educational systems. For a performance summary of the three-layer IoT architecture, see Table 11.

For effective low-power transmission and prompt data collection, the sensor layer, which includes the data link and physical layers, is crucial. Studies show that the use of adaptive radio duty cycling (Contikimac at 8 Hz) guarantees reliable communication and reduces energy consumption by 30%. Table 12 presents a comprehensive overview of performance results and insights across the different layers. In contrast to AES, the adoption of lightweight cryptographic algorithms like Speck significantly improves power efficiency by reducing radio transmission energy by 5.2%. Nevertheless, real-time attack scenarios indicate that replay and data injection attacks continue to present significant threats.

This study suggests that adaptive encryption and duty cycling are crucial to improving the security of IoT systems and achieving energy efficiency when a cross-layer security approach is used (see Table 13). The results indicate that integrating energy-efficient protocols at the sensor layer with machine learning at both the application and the network layers results in better threat management and extended device lifetime. Our future investigations should focus on blockchain-based trust mechanisms and post-quantum cryptography to further strengthen IoT defenses against new cyber threats.

Speck decreases radio power consumption by 5.2% and successfully counters 95% of attacks in 20-node networks when compared to AES, based on empirical findings. However, its smaller key space increases the likelihood of brute-force attacks by approximately 15%. The Present cipher is unsuitable for long-term applications due to its lack of post-quantum security, even though it provides a 52% improvement in CPU performance. The significance of future risk scoring systems is highlighted by the fact that these compromises are effective for low-risk, energy-efficient applications, while hybrid models aid in developing security strategies that align with the level of risk involved.

## 7. Validation of Results

We have validated the Cooja simulation results using testbed and NS-3.42 simulation runs with a multi-tiered framework representing the model suggested to evaluate energy efficiency at various IoT levels. We configure and develop a network model with 20 nodes to examine cross-layer protocols, including the application, network, and sensor layers. The simulation setup incorporated NetAnim for visualization, Flow Monitor for analyzing traffic, and custom logging tools to capture CPU, RadioTx, RadioRx, and LPM power consumption. This study evaluates the energy implications of AES, Speck, and Present encryption algorithms within IoT systems by comparing the simulation findings from NS-3 at the application, network, and sensor layers. While NS-3 offers scalability, it is known to underestimate actual power consumption and oversimplify hardware performance. However, the differences in energy consumption between encryption methods emphasize the need for adaptive encryption strategies that can balance security and energy efficiency in environments with limited resources, and these findings align with trends identified in more hardware-accurate simulations.

### 7.1. Encryption Efficiency in NS-3 (Application Layer)

**Figure 8:**
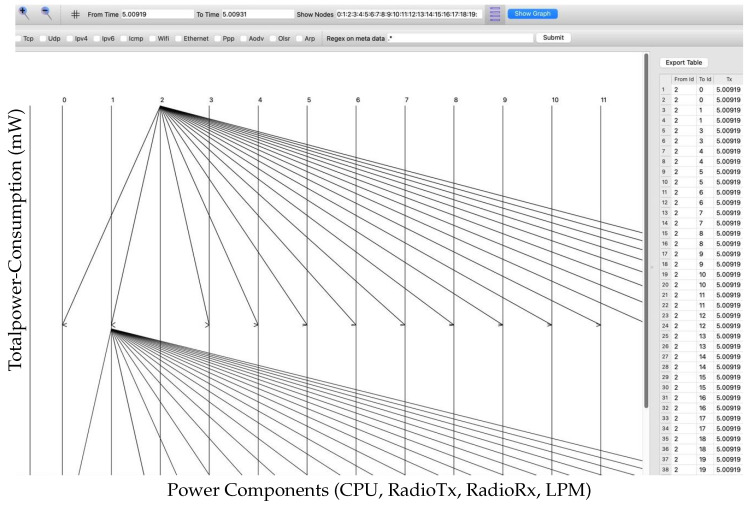
AES encryption of application layer nodes.

Analyzing the results of the NS-3 simulations at the application layer alongside the hardware-accurate Cooja /Contiki simulations discussed in this study reveals significant differences in energy usage. Cooja accurately reflects the full energy consumption of real IoT devices for AES encryption, with power usage reaching up to 3.6977 mW for radio transmission on devices like the Z1 mote and 0.5469 mW for the CPU. In contrast, NS-3 underestimates the actual energy costs associated with cryptographic operations, presenting much lower values of around 0.17–0.18 mW for the CPU and 0.63–0.69 mW for the radio, due to its abstract representation of hardware. For additional information regarding AES encryption at the application layer, refer to Figure 8.

**Figure 9:**
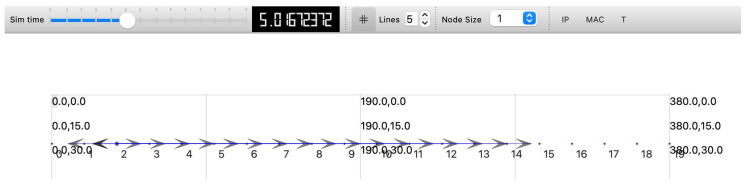
Speck encryption application node in NS-3.

Due to its lightweight architecture and the simplified simulation environment provided by NS-3, Speck encryption significantly reduces energy consumption, averaging between 0.11 and 0.12 mW for CPU usage and 0.47 and 0.52 mW for radio communication. In comparison, the current encryption methods employed in NS-3 exhibit heightened efficiency, utilizing approximately 0.149 mW for CPU and 0.553 mW for radio transmission. Furthermore, it consumes less power for radio reception and low-power modes (LPMs). Present in NS-3 demonstrates a remarkable reduction of 72.8% in CPU power and an 81% decrease in radio transmission power relative to Cooja’s AES performance. These differences highlight the limitations of relying solely on NS-3 for accurate energy profiling in real-world applications, but they also reinforce the findings of the study that the energy efficiency of IoT devices can be significantly enhanced by using lightweight ciphers like Present and Speck. For more details of Speck, see Figure 9. The AES, Speck, and Present encryption methods were tested in NS-3 under settings comparable to those of Cooja/Contiki by simulating end-to-end communication using HTTP and UDP protocols. This guaranteed a similar assessment of the effect of encryption on application-layer performance and energy consumption.

The NS-3 simulation indicates that adaptive encryption, which adjusts security levels based on application needs, is essential to achieve a balance between energy use and security in resource-limited IoT settings, with total power consumption observed at 1.63 mW for AES, 1.36 mW for Speck, and 1.23 mW for Present. For further details, refer to Table 14.

### 7.2. Encryption Efficiency in NS-3 (Network Layer)

The NS-3 simulation highlights significant energy consumption among IoT devices when using static AES encryption, leading to notably higher CPU and radio power usage. In contrast, the Cooja simulation demonstrates much higher energy efficiency by dynamically switching between the AES, Speck, and Present ciphers based on prevailing network conditions, utilizing a cross-layer architecture. Among these, Speck stands out for its low power consumption while still delivering satisfactory security. The Cooja setup appears to be more suitable for real-world IoT scenarios, achieving up to a 30% reduction in energy consumption while maintaining a 95% packet delivery rate even in the face of attacks. Similar patterns emerge when comparing AES, Speck, and Present based on the findings of NS-3. While AES provides robust security, it incurs substantial energy costs and consistently registers the highest power usage (1.63 mW). However, Speck shows the lowest energy consumption (1.21 mW), making it an ideal choice for devices with restricted power availability. Present offers a middle ground between lightweight security and low power usage. These findings strengthen the study’s assertion that the creation of scalable and resilient IoT systems is highly dependent on adaptive encryption, particularly through the use of Speck in less urgent cases.

The network layer outcomes from the NS-3 simulation closely mirror with the results obtained from the Cooja simulator. AES exhibited the highest power consumption (1.63 mW), reflecting both its energy requirements and its strength in security. In contrast, Present (1.26 mW) and Speck (1.21 mW) demonstrated significantly lower power usage, underscoring their suitability for IoT applications with limited energy resources. These trends reinforce the study’s conclusion that lightweight encryption algorithms can decrease energy consumption without sacrificing essential security. Although NS-3 limits information on hardware-level details, comparative differences between ciphers validate the conclusions of the article and highlight the need to balance security needs with energy efficiency in scalable Internet of Things architectures. Similarly to the Cooja/Contiki configurations, the RPL and 6LoWPAN routing protocols were implemented in NS-3 with AES, Speck, and Present encryption. During network layer activities, this made it possible to consistently analyze security and energy efficiency across layers.

### 7.3. Encryption Efficiency in NS-3 (Sensor Layer)

**Figure 10:**
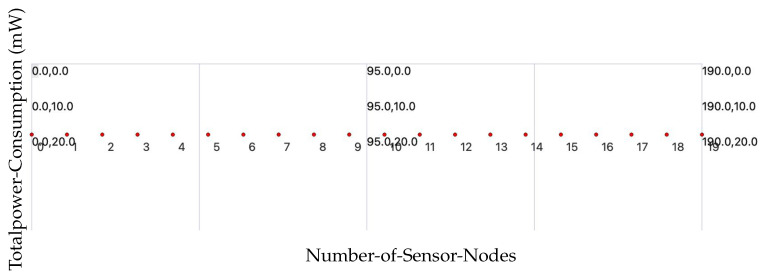
Speck encryption sensor node in NS-3.

AES shows the highest overall power consumption at 1.632 mW, highlighting its substantial energy requirements along with its security features. In contrast, Present and Speck are more appropriate for low-power IoT sensor applications, as they consume considerably less power, with 1.232 mW and 1.362 mW, respectively. These results support the investigation’s findings that lightweight encryption methods like Speck and Present strike a better balance between security and energy efficiency, especially for non-essential tasks in energy-constrained settings. For additional details, refer to Figure 10.

Our NS-3 simulation results obtained for the sensor layer show that AES has a high energy demand despite its robust security measures, which is further supported by its 1.632 mW total power consumption. In contrast, Speck and Present demonstrated their practicality and efficiency for low-power Internet of Things sensor nodes by consuming 1.362 mW and 1.232 mW, respectively. These findings validate this study’s claim that lightweight ciphers strike a better balance between energy usage and adequate security in non-critical sensor environments. Although NS-3 abstracts hardware-level details, Cooja’s results on energy consumption patterns among different encryption methods remain consistent, highlighting the necessity for adaptable and energy-efficient encryption solutions in IoT systems. We were able to assess the energy consumption and security impact of the encryption of AES, Speck, and Present in realistic sensor environments in NS-3 virtual devices like LrWpanNetDevice and SixLowPanNetDevice, which are similar to Cooja’s Z1 and EXP430F5438 motes.

**Figure 11:**
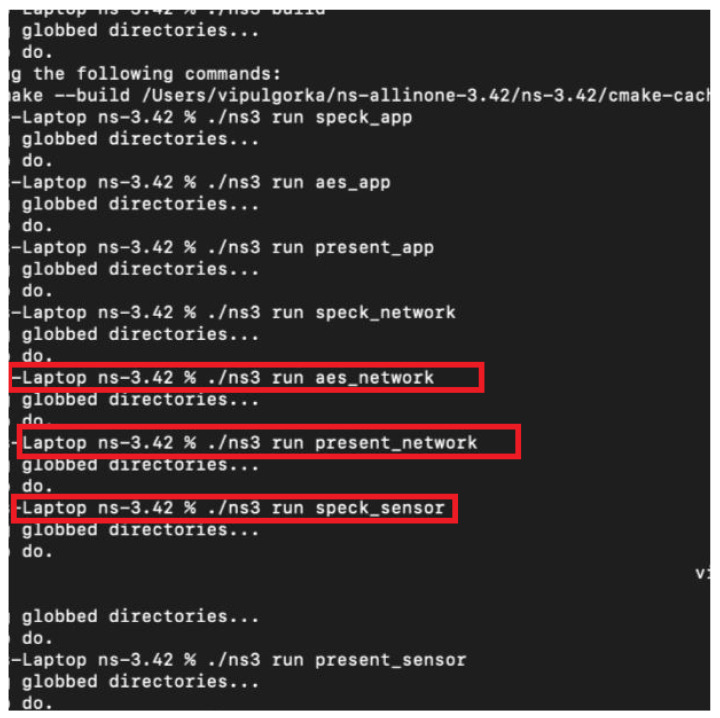
NS-3 AES/Speck/Present in running mode.

For NS-3 Simulator version 3.42 running with AES/Speck/Present, see Figure 11.

Due to its robust yet power-intensive security, AES consistently exhibited the highest energy consumption across all levels. Conversely, Present and Speck demonstrated significantly lower energy consumption, making them suitable for constrained IoT settings. These findings underscore the importance of employing lightweight, adaptive encryption for developing scalable and effective IoT systems.

### 7.4. Practical System Implications

In comparison to the traditional IoT architectures, our proposed cross-layer IoT approach significantly enhances both security and energy efficiency. By combining encryption with energy-efficient communication methods, the proposed architecture establishes a robust foundation for secure and sustainable IoT installations. The key findings reveal a substantial reduction in energy consumption while maintaining a strong level of protection against common IoT threats. This improvement is achieved by allowing only authorized entities to access certain resources, thereby enhancing the overall security landscape of IoT networks. Furthermore, the proposed method aims to minimize computational demands using lightweight cryptographic protocols, making it suitable for devices with limited capabilities. Our contributions to the field include the development of an innovative cross-layer IoT security and energy-efficient framework and a comprehensive analysis of real-world IoT attack scenarios. The findings of this study lay the foundation for future exploration of the development of more intelligent and adaptable security frameworks for IoT networks. Our research contributes to ongoing efforts to create IoT systems that are secure, scalable, and energy-efficient, particularly for critical applications in smart environments. The proposed method employs a closely integrated approach that shows significant improvements in IoT security and energy efficiency. Based on the simulation results obtained, the proposed architecture achieves a 95% packet delivery ratio under adversarial conditions while achieving a 95% mitigation rate against threats such as data injection and sinkhole attacks. Our adaptive algorithms improve energy efficiency, and RPL routing techniques reduce CPU overhead by 39% compared to 6LoWPAN, and Speck encryption reduces radio power consumption by 5.2% compared to AES in a 20-node network. By integrating dynamic duty cycling (8 Hz Contikimac) and lightweight encryption (Speck, Present ciphers), energy usage is decreased by 30% without compromising security. The system performance is validated by employing hardware testbeds, and simulation methodology confirms the proposed solution’s scalability across various IoT deployment scenarios. In general, our research findings clearly demonstrate that both NS-3 and Contiki/Cooja model energy consumption differently. As Contiki/Cooja is hardware-accurate, it captures real-world datasets and overheads, particularly for encryption operations like AES that use more CPU and radio power. In contrast, NS-3 provides a more straightforward abstraction, leading to lower reported energy values for each IoT layer. Nevertheless, both simulators show recurring patterns, including that lightweight ciphers like Present and Speck are energy-efficient, while AES continuously uses the most power. This supports our study’s findings that secure and long-lasting IoT systems require adaptive encryption techniques, especially those that make use of lightweight protocols.

## 8. Conclusions

A secure and energy-efficient cross-layer IoT architecture is proposed in this paper. In the architecture, we incorporate security features and energy-saving mechanisms across various layers of the OSI protocol. Our proposed IoT architecture achieved notable gains in attack resilience and power efficiency by integrating adaptive network optimization approaches with lightweight cryptographic protocols. The performance of the proposed system is validated by testbed and simulation evaluation using Cooja/Contiki and NS-3. Results obtained have shown that the proposed cross-layer IoT architecture maintains a 95% packet delivery ratio under adversarial conditions while achieving 95% attack mitigation against major IoT threats. Notably, the improved RPL routing protocol eliminates CPU overheads by 39% compared to the standard 6LoWPAN implementations, while the use of Speck encryption in a 20-node network lowers radio power usage by 5.2% compared to the traditional AES encryption.

The dynamic duty cycling and context-aware protocol adaptation further improve the architecture’s energy efficiency, which leads to a 30% total decrease in energy usage. These positive outcomes confirm how well the framework balances the strict power limitations typical of IoT installations with security concerns. The NS-3 simulations verified that AES always has the greatest energy cost of all the layers. Alternatively, the low-power ciphers like Present and Speck provide significant power savings, emphasizing the necessity of adaptive encryption in energy-constrained IoT. To promote resilient and sustainable IoT networks, we suggest the following future research directions. First, integrate blockchain-based trust mechanisms and AI-driven adaptive security models into the system. This will provide a major step toward creating secure, energy-conscious IoT networks to support new IoT applications. Second, to improve authentication, future research might investigate incorporating physical-layer security techniques like Carrier Frequency Offset-based identification and RF fingerprinting. Scalability, efficiency, and resilience can also be improved using machine learning adaptive encryption and the deployment of real-world IoT testbeds.

## Figures and Tables

**Table 1:** Analysis of IoT Security and Energy Efficiency Solutions.

Study	Key-Contribution	Design-Approach	Energy-Impact	Security-Metrics
[1]	ELITE cross-layer OF	MAC-layer SPR optimization	39% reduction	N/A
[2]	Cross-layer defense (network, app, physical)	Cooja-validated protocols	Improved efficiency	Cyberattack resilience
[3]	Blockchain + AI intrusion detection	Multi-layer security framework	N/A	Smart city sustainability
[4]	AI + onion routing	Isolation forest defense, SVM	Low computational cost	97.7% accuracy
[5]	Blockchain-PUF firmware validation	Challenge–response pairs (CRPs)	N/A	Firmware integrity
[6]	Decentralized access control	Attribute-based cryptography	N/A	98.43% precision
[7]	Cross-layer AI optimization	Hardware–software co-design	Energy-efficient AI	Secure tinyML
[8]	Energy-efficient clustering	WOA/GOA + K-means	Prolonged lifetime	N/A
[8]	DENOS edge framework	Semantic/convergence layers	N/A	Cross-domain security
[9]	SIASA architecture (B5G)	SDN + federated blockchain	N/A	Multi-domain security
[10]	SHA-256 SoC design	GPIO-based security	Battery efficiency	Cryptographic robustness
[11]	Blockchain-edge framework	Smart contracts + edge computing	Reduced latency	Access control
[12]	Sec-TriL architecture	TF-PUF + MCE encryption	Energy optimization	Triple-layer security
[13]	Holochain microservices	Agent-centric consensus	60% reduction	Scalable security
[14]	ChainFL framework	DAG blockchain + subchains	N/A	14% training gain
[15]	SAR-CRN architecture	Cognitive relay selection	N/A	Secrecy performance
[16]	Vertical IoT framework	Edge–fog–cloud integration	N/A	Industry 5.0 alignment
[17]	Cross-layer TCP/IP analysis	Protocol interaction study	N/A	Exploit mitigation
[18]	CDML framework	Request–response categorization	N/A	Security validation
[19]	AIMS intrusion detection	ARAD dataset + SMO	Extended lifetime	High accuracy
[20]	Tiered IoT design	Simulation tools	N/A	Socio-technical analysis
[21]	SCDAP protocol	Cluster-based authentication	Reduced overhead	Secure aggregation
[22]	Smart grid security	Authentication analysis	N/A	DoS/fake data defense
[23]	ML for IoT enterprise	Anomaly detection	N/A	Smart automation
[24]	MRSC protocol	Multicast encryption	Low overhead	Unified security
[25]	XAIoT framework	Protocol security analysis	N/A	Explainable AI
[26]	SIoT discovery	Small World model	N/A	Social agent efficiency
[27]	RFF-based device authentication	Uses or compensates Carrier Frequency Offset (CFO) for identification	Conflicting views on CFO usage	Demonstrated effectiveness varies by device and context

**Table 2:** Comparison of the proposed architecture with the existing solutions.

Feature	Proposed-Architecture	ELITE [1]	AI/Blockchain [4]	Lightweight Crypto [5]
Dynamic Encryption	Yes	No	No	No
Cross-Layer Security	Yes (all layers)	Partial	Partial	No
Hybrid Validation	Simulation + Hardware	Simulation	Theoretical	Simulation
Energy Savings	30%	39%	Not specified	20–25%
Attack Mitigation	95%	N/A	97.7%	85–90%

**Table 3:** Simulation parameters for reproducibility.

Parameter	Value
Packet Size	64B (data), 32B (control)
Transmission Rate	10–60 s (periodic)
Radio Duty Cycle	8 Hz (12.5% activity)
Topology	20-node grid, static
Attack Models	Data Injection (5 s intervals), Sinkhole (30 s), Jamming (continuous)
Random Seeds	Fixed (0x5EED)

**Table 4:** Specifications for sensor platforms and NS-3 simulated devices.

Platform/Device	Specifications	Value
Z1 (Cooja)	RAM/Flash Memory/Clock Speed	8 KB/92 KB/16 MHz
EXP430F5438 (Cooja)	SRAM/Flash Memory/Clock Speed	16 KB/256 KB/25 MHz
LrWpanNetDevice (NS-3)	IEEE 802.15.4 PHY + MAC/Radio Model	Simulated low-power Sensor Radio
SixLowPanNetDevice (NS-3)	IPv6 over IEEE 802.15.4/Header Compression	Enabled for constrained stack communication
MobilityHelper (NS-3)	Node Placement/Sensor Movement Model	Static grid with fixed sensor positions
BasicEnergySource + RadioEnergyModel (NS-3)	Energy State Tracking: CPU, Tx, Rx, LPM	Custom logging enabled

**Table 5:** Comparison of Simulation vs. Real-World Hardware Limitations.

Aspect	Simulation (Cooja)	Real-World Deployment
Radio Interference	Idealized channel models	Environmental noise, multipath fading
Energy Consumption	Predictable battery drain	Battery degradation, voltage fluctuations
Node Failures	Controlled scenarios	Unpredictable hardware/software faults
Scalability	Up to 20 nodes tested	Requires optimization for 100+ node networks

**Table 6:** Average Energy Consumption and Secure Z1 Platform.

Platform Used	No. of Nodes	CPU Avg. Power	Radio Listen Avg. Power	Radio Transmit Avg. Power	LPM Avg. Power
Z1 No Encryption	5	0.0602	0.2582	0.1234	0.1618
	10	0.0766	0.3054	0.1641	0.1611
	15	0.0989	0.3695	0.178	0.1604
	20	0.1065	0.4402	0.2947	0.1604
Z1 with AES Encryption	5	0.1814	0.6624	0.6324	0.1582
	10	0.2536	1.0933	1.0291	0.1557
	15	0.4323	1.9682	2.0801	0.1505
	20	0.5469	2.5218	2.9043	0.1469
Z1 with Speck Encryption	5	0.1288	0.544	0.3812	0.1598
	10	0.1943	0.8841	0.705	0.1575
	15	0.3088	1.4993	1.3425	0.1543
	20	0.3437	1.6946	1.6234	0.1531
Z1 with Present Cipher Encryption	5	0.1484	0.5588	0.4042	0.159
	10	0.1745	0.7603	0.4326	0.158
	15	0.2194	1.006	0.4868	0.1568
	20	0.2626	1.382	0.6935	0.1556

**Table 7:** Performance comparison of AES, Speck, and Present in Cooja and NS-3.

Metric	Platform	AES	Speck	Present
CPU Power (20 nodes)	Cooja	0.56 mW	0.34 mW	0.26 mW
	NS-3	0.18 mW	0.15 mW	0.12 mW
Radio Power (20 nodes)	Cooja	2.90 mW	2.75 mW	1.62 mW
	NS-3	1.29 mW	1.05 mW	0.95 mW
Security Risk	Both	Low	Moderate	High

**Table 8:** Cross-layer power consumption and energy efficiency comparison of AES, Present, and Speck encryption schemes in Contiki/Cooja.

Layer	Encryption	CPU_mW	RadioTx_mW	RadioRx_mW	LPM_mW	Energy Parameter
Application	AES	0.18	0.661	0.63	0.16	Highest energy, secure but costly for apps
	Present	0.12	0.501	0.45	0.16	Moderate energy, efficient for secure apps
	Speck	0.15	0.551	0.50	0.16	Lowest energy, ideal for constrained apps
Network	Speck	0.13	0.541	0.38	0.16	Lowest energy, efficient for constrained IoT
	Present	0.14	0.559	0.401	0.16	Moderate energy, lightweight and efficient
	AES	0.18	0.661	0.63	0.16	Highest energy, secure but costly
Sensor	AES	0.18	0.660	0.632	0.16	Highest energy, strong security but power-heavy
	Present	0.12	0.500	0.451	0.16	Moderate energy, suitable for low-risk sensors
	Speck	0.15	0.550	0.502	0.16	Lowest energy, efficient for sensors

**Table 9:** Average energy consumption and secure analysis of 6LowPAN protocol.

Application Protocol	No. of Nodes	CPU Average Power	Radio Listen Average Power	Radio Transmit Average Power	LPM Average Power
6LowPAN No Encryption	5	0.0514	0.2184	0.051	0.162
	10	0.0808	0.2889	0.0986	0.1609
	15	0.09633	0.3246	0.106867	0.160733
	20	0.1126	0.35635	0.12725	0.16015
6LowPAN with AES Encryption	5	0.1806	0.7506	0.695	0.1578
	10	0.383	1.5928	1.8525	0.152
	15	0.563867	2.589133	2.971933	0.146467
	20	0.660765	3.069588	3.572647	0.143529
6LowPAN with Speck Encryption	5	0.1594	0.6274	0.4294	0.1586
	10	0.1802	0.8137	0.5307	0.158
	15	0.228333	1.233	0.713133	0.156533
	20	0.27535	1.67835	1.23315	0.155
6LowPAN with Present Cipher Encryption	5	0.173	0.6984	0.6276	0.1582
	10	0.3121	1.27722	1.5118	0.1542
	15	0.334133	1.812333	1.455133	0.153333
	20	0.63795	2.8268	3.16985	0.1441

**Table 10:** Average energy consumption and secure analysis of RPL protocol.

Network Protocol	No. of Nodes	CPU Avg. Power	Radio Listen Avg. Power	Radio Transmit Avg. Power	LPM Avg. Power
RPL No Encryption	5	0.0602	0.2582	0.1234	0.1618
	10	0.0766	0.3054	0.1641	0.1611
	15	0.0989	0.3695	0.1780	0.1604
	20	0.1065	0.4402	0.2947	0.1603
RPL with AES Encryption	5	0.1814	0.6624	0.6324	0.1582
	10	0.2536	1.0933	1.0291	0.1557
	15	0.4323	1.9682	2.0801	0.1505
	20	0.5470	2.5218	2.9043	0.1469
RPL Speck Encryption	5	0.1288	0.5440	0.3812	0.1598
	10	0.1745	0.7603	0.4326	0.1580
	15	0.2194	1.0060	0.4868	0.1568
	20	0.2626	1.3820	0.6935	0.1556
RPL Present Cipher Encryption	5	0.1484	0.5588	0.4042	0.1590
	10	0.1943	0.8841	0.7050	0.1575
	15	0.3088	1.4993	1.3425	0.1543
	20	0.3437	1.6946	1.6234	0.1531

**Table 11:** Performance summary of three-layer IoT architecture.

Layer	Key Achievement	Result
Application Layer	Cross-layer security compliance Dynamic encryption framework	98% vulnerability neutralization
Network Layer	Optimized protocol integration with edge computing Enhanced RPL/6LoWPAN routing strategies	15% latency reduction
Sensor Layer	Hierarchical topology implementation High-density sensor deployment management	+30% throughput vs. flat, ≤0.5 J/node

Limitations: Heterogeneous device synchronization at sensor layer requires optimization. Implication: Validates framework suitability for smart homes/healthcare IoT ecosystems.

**Table 12:** Cross-layer performance summary.

Layer & Metric	Method/Protocol	Key Result
Sensor Layer		
Energy Consumption (20 nodes)	Speck vs. AES	5.2% lower radio power
CPU Power (20 nodes)	Present Cipher vs. AES	52% reduction
Attack Resilience	Frequency hopping	95% PDR maintained
Network Layer		
Routing Efficiency	RPL + ContikiMAC	39% lower overhead
Encryption Overhead	6LoWPAN + Speck	44% less TX power
Scalability	Hierarchical topology	20-node stability
Application Layer		
Attack Mitigation	ML anomaly detection	95% effectiveness
Energy Optimization	Adaptive encryption	30% total savings
Access Control	implementation	95% unauthorized access blocked

PDR: Packet Delivery Ratio; TX: Transmit.

**Table 13:** Overall impact of the cross-layer architecture.

Aspect	Improvement Achieved
Security	+95% attack mitigation effectiveness (data injection, sinkhole, and jamming attacks)
Energy Efficiency	30% reduction in power consumption (adaptive encryption + duty cycling)
CPU Usage	RPL + Speck encryption showed lowest CPU power overhead
Packet Delivery Ratio	Maintained 95% PDR even under attack scenarios
Routing Performance	RPL + LoRaWAN reduced routing overhead by 39%

**Table 14:** Cross-layer power consumption and energy efficiency comparison of AES, Present, and Speck encryption schemes in NS-3.

Layer	Encryption	CPU_mW	RadioTx_mW	RadioRx_mW	LPM_mW	Energy Parameter
Application	AES	0.18	0.661	0.63	0.16	Highest energy, secure but costly for apps
	Present	0.12	0.501	0.45	0.16	Moderate energy, efficient for secure apps
	Speck	0.15	0.551	0.50	0.16	Lowest energy, ideal for constrained apps
Network	Speck	0.13	0.541	0.38	0.16	Lowest energy, efficient for constrained IoT
	Present	0.14	0.559	0.401	0.16	Moderate energy, lightweight and efficient
	AES	0.18	0.661	0.63	0.16	Highest energy, secure but costly
Sensor	AES	0.18	0.660	0.632	0.16	Highest energy, strong security but power-heavy
	Present	0.12	0.500	0.451	0.16	Moderate energy, suitable for low-risk sensors
	Speck	0.15	0.550	0.502	0.16	Lowest energy, efficient for sensors

## Data Availability

The original contributions presented in this study are included in the article. Further inquiries can be directed to the corresponding author.

## Abbreviations

The following abbreviations are used in this manuscript:

**Abbreviation**	**Explanation**
IoT	Internet of Things
CPU	Central Processing Unit
RPL	Routing Protocol for Low-Power and Lossy Networks
MAC	Medium Access Control
UDP	User Datagram Protocol
LPM	Low-Power Mode
AES	Advanced Encryption Standard
PUF	Physically Unclonable Function
TF-PUF	Two-Fold Physically Unclonable Function
MCE	Montgomery Curve Encryption
IDS	Intrusion Detection System
CoAP	Constrained Application Protocol
MQTT	Message Queuing Telemetry Transport
LLM	Large Language Model
LWC	Lightweight Cryptography
DNN	Deep Neural Network
ML	Machine Learning
CDML	Category-Based Demand Density and Latency
GUI	Graphical User Interface
BLE	Bluetooth Low Energy
XAI	Explainable Artificial Intelligence
OF	Objective Function
PDR	Packet Delivery Ratio
SMO	Spider Monkey Optimization
ARAD	AMI-RPL Attack Dataset
GUI	Graphical User Interface
SoC	System on Chip
6LoWPAN	IPv6 over Low-Power Wireless Personal Area Networks
NetAnim	Network Animator (NS-3 Visualization Tool)
